# Formation of nuclear condensates by the Mediator complex subunit Med15 in mammalian cells

**DOI:** 10.1186/s12915-021-01178-y

**Published:** 2021-11-17

**Authors:** Yuanyuan Shi, Jian Chen, Wei-Jie Zeng, Miao Li, Wenxue Zhao, Xing-Ding Zhang, Jie Yao

**Affiliations:** 1 Molecular Cancer Research Center, School of Medicine, Shenzhen Campus of Sun Yat-sen University, Sun Yat-sen University, Shenzhen, China; 2 Present Address: Allen Institute for Cell Science, Seattle, WA 98109 USA

**Keywords:** Nuclear condensates, Mediator, Med15, Transcription, Cell imaging

## Abstract

**Background:**

The Mediator complex is an evolutionarily conserved multi-subunit protein complex that plays major roles in transcriptional activation and is essential for cell growth, proliferation, and differentiation. Recent studies revealed that some Mediator subunits formed nuclear condensates that may facilitate enhancer-promoter interactions and gene activation. The assembly, regulation, and functions of these nuclear condensates remain to be further understood.

**Results:**

We found that Med15, a subunit in the tail module of the Mediator complex, formed nuclear condensates through a novel mechanism. Nuclear foci of Med15 were detected by both immunostaining of endogenous proteins and live cell imaging. Like Med1 foci and many other biomolecular condensates, Med15 foci were sensitive to 1, 6-Hexanediol and showed rapid recovery during fluorescence recovery after photobleaching. Interestingly, overexpressing DYRK3, a dual-specificity kinase that controls the phase transition of membraneless organelles, appeared to disrupt Med1 foci and Med15 foci. We identified two regions that are required to form Med15 nuclear condensates: the glutamine-rich intrinsically disordered region (IDR) and a short downstream hydrophobic motif. The optodroplet assay revealed that both the IDR and the C-terminal region of Med15 contributed to intracellular phase separation.

**Conclusions:**

We identified that the Mediator complex subunit Med15 formed nuclear condensates and characterized their features in living cells. Our work suggests that Med15 plays a role in the assembly of transcription coactivator condensates in the nucleus and identifies Med15 regions that contribute to phase separation.

**Supplementary Information:**

The online version contains supplementary material available at 10.1186/s12915-021-01178-y.

## Background

Membraneless organelles are specialized subcellular compartments that enrich an ensemble of macromolecules and play important roles in cell physiology. Some well-characterized examples of membraneless organelles include the nucleolus, nuclear speckles, Cajal bodies, and stress granules. Assembly of membraneless organelles often involves liquid-liquid phase-separation (LLPS), a process that is regulated by multivalency and weak interactions among the protein and RNA constituents and is concentration-dependent [1, 2]. Recent studies have started to reveal the underlying principles of LLPS and suggested that LLPS may serve as a fundamental mechanism linking cell physiology and disease [3–5]. Proteins that undergo LLPS often contain intrinsically disordered regions (IDRs) consisting of low-complexity amino acid sequences [6–8]. Many membraneless organelles are dissolved during mitosis through actions of protein kinases including DYRK3 [9]. Despite many previous studies, much remains to be discovered on the formation, regulation, and functions of membraneless organelles in cells.

Recent findings on biomolecular condensates formed by eukaryotic transcription machineries suggested a role of phase separation in gene expression [10]. For example, biomolecular condensates were observed to form in vitro and in vivo by the C-terminal domain of RNA polymerase II (Pol II) [11, 12], the Mediator complex [13, 14], or by multiple sequence-specific transcription factors (TFs) containing disordered amino acid regions at their activation domains [6, 15]. Furthermore, diverse TFs can form phase-separated condensates with Mediator, suggesting that nuclear condensates may function in gene activation [15]. These new findings complement the conventional view of eukaryotic gene regulation that Mediator transduces signals from enhancer-bound TFs to the core transcriptional machinery [16]. Further studies are needed to better understand the mechanisms of condensate formation and their proposed functions in gene expression.

The Mediator complex is an evolutionarily conserved multi-subunit transcription coactivator complex that is essential for growth and survival of all cells [16]. Mediator has a flexible structure and is organized into head, middle, tail, and Cdk8 modules [17]. Recent studies found that the Mediator subunit Med1 formed nuclear foci in mouse embryonic stem cells [13] and yeast Med15 protein could form condensates with transcription activator GCN4 in vitro [15]. Med1 and Med15 belong to the middle module and the tail module of the Mediator complex, respectively. Med15 contains a glutamine-rich IDR and interacts with multiple transcription activators through its structured or unstructured domains [18–20]. Previous work indicated that Med3 and Med15 could form amyloid-like aggregates in yeast cells upon H_2_O_2_ stress [21], but whether Med15 forms condensates in mammalian cells is unclear.

In this work, we provided experimental evidence that Med15 forms nuclear condensates in mouse and human cells. Med15 foci mimicked Med1 foci in the sensitivity to 1, 6-Hexanediol and rapid FRAP recovery. Med1 foci and Med15 foci were largely abolished in mitotic cells and upon overexpressing DYRK3 kinase. Interestingly, formation of Med15 nuclear condensates required both its glutamine-rich IDR region and a short sequence of hydrophobic amino acids. Upon blue light induction, either the N-terminal IDR or the C-terminal region of Med15 was sufficient to form optodroplets in living cells. We also found concentration-dependent effects of 1, 6-Hexanediol on the activation of immediate early genes upon serum stimulation.

## Results

### Med1 nuclear foci are sensitive to 1, 6-Hexanediol and are dissolved during mitosis

**Fig. 1:**
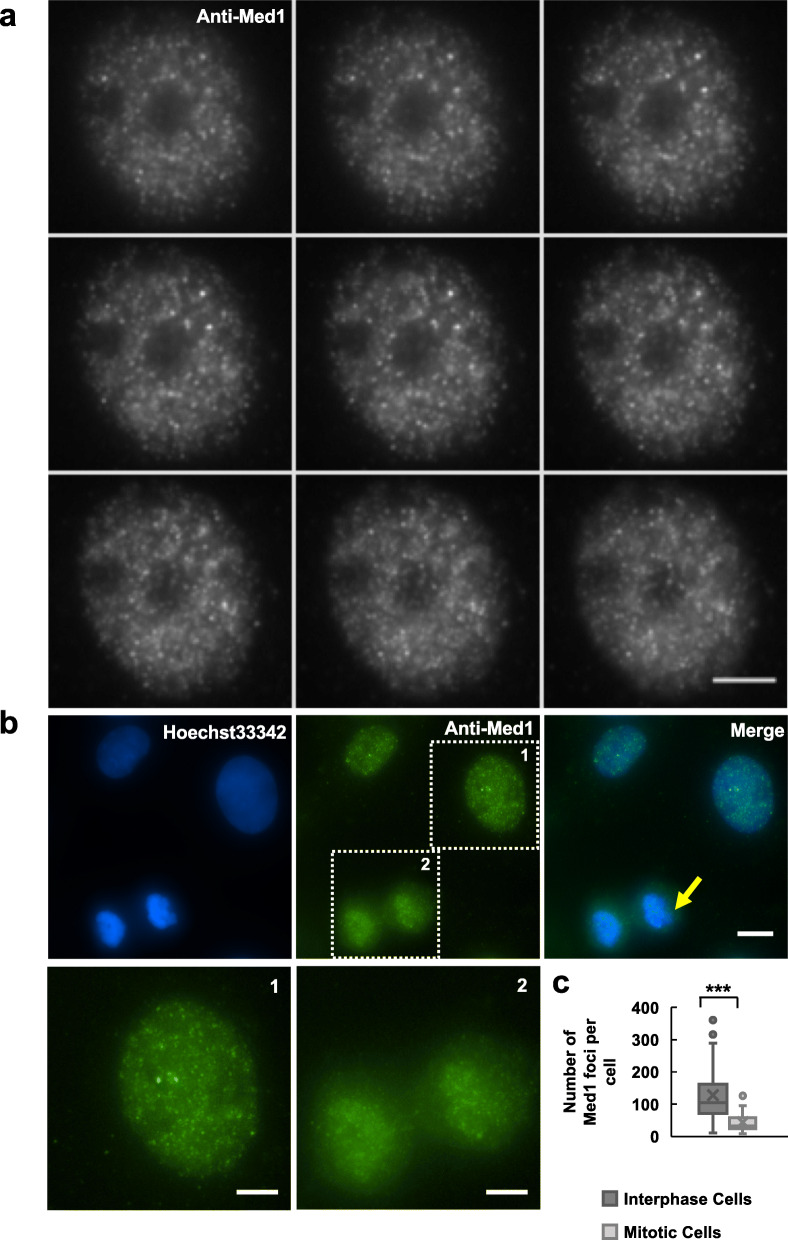
Distributions of Med1 nuclear foci in interphase and mitotic human cells. **a** Wide-field fluorescence images of a human U2OS cell nucleus stained with an anti-Med1 antibody. The *z*-interval between individual images is 0.3 μm. Scale bar: 5 μm. **b** Interphase and mitotic U2OS cells co-stained with an anti-Med1 antibody (green) and Hoechst33342 (blue). Scale bar: 10 μm. The yellow arrow indicates a cell undergoing mitosis. Insets 1 and 2 show the enlarged images of Med1 foci in interphase and mitotic cells, respectively. Scale bars: 5 μm. Similar results were obtained from three independent experiments. **c** The number of Med1 foci quantified in individual interphase or mitotic U2OS cells by AirLocalize program [22] (Intensity threshold: 450). The numbers of analyzed cells were 46 and 17, respectively. Student’s *t* test: *p* < 0.001 (indicated by ***)

Immunofluorescence staining revealed that Med1 formed numerous, well-distinguishable nuclear foci in U2OS cells (Fig. 1a), consistent with results with imaging GFP-Med1 in CRISPR knock-in mouse embryonic stem (ES) cells [13]. The specificity of this antibody against endogenous Med1 proteins was confirmed by western blot (Additional file 1: Fig. S1a). Next, we performed Med1 immunostaining in cells treated with 1, 6-Hexanediol, an aliphatic alcohol that has been frequently used to study biomolecular condensates in cells. Distinct Med1 foci became invisible in most cells after 1 min of Hexanediol treatment (Additional file 1: Fig. S1b). Med1 foci reappeared in most cells at 10 min and 30 min after Hexanediol withdrawal (Additional file 1: Fig. S1b). Because Med1 foci could be distinguished as individual fluorescence spots in a 3D image stack (Fig. 1a), we used the AirLocalize program [22] to obtain the number of Med1 foci in cells. The median number of Med1 foci per nucleus decreased from ~ 150 in untreated cells to less than 50 in Hexanediol-treated cells and increased to 200–300 in cells recovered for 10 min and 30 min (Additional file 1: Fig. S1c). We note that fluorescence intensities of Med1 foci were generally higher in recovered cells than in untreated cells (Additional file 1: Fig. S1b), which might explain the higher number of Med1 foci in recovered cells because the same intensity threshold was used for quantification.

Many well-known membraneless organelles, such as nuclear speckles, nucleoli, and Cajal bodies, are dissolved during mitosis [23–25]. Nonetheless, the status of Med1 nuclear foci in mitotic cells has not been described. By immunofluorescence staining, we found a more homogeneous localization of Med1 in mitotic cells than in interphase cells (Fig. 1b). The median number of Med1 foci decreased from ~ 150 in interphase cells to less than 50 in mitotic cells (Fig. 1c). Thus, Med1 nuclear foci resemble other membraneless organelles in their ability to dissolve during mitosis.

### Characterization of nuclear condensates formed by Med15

**Fig. 2:**
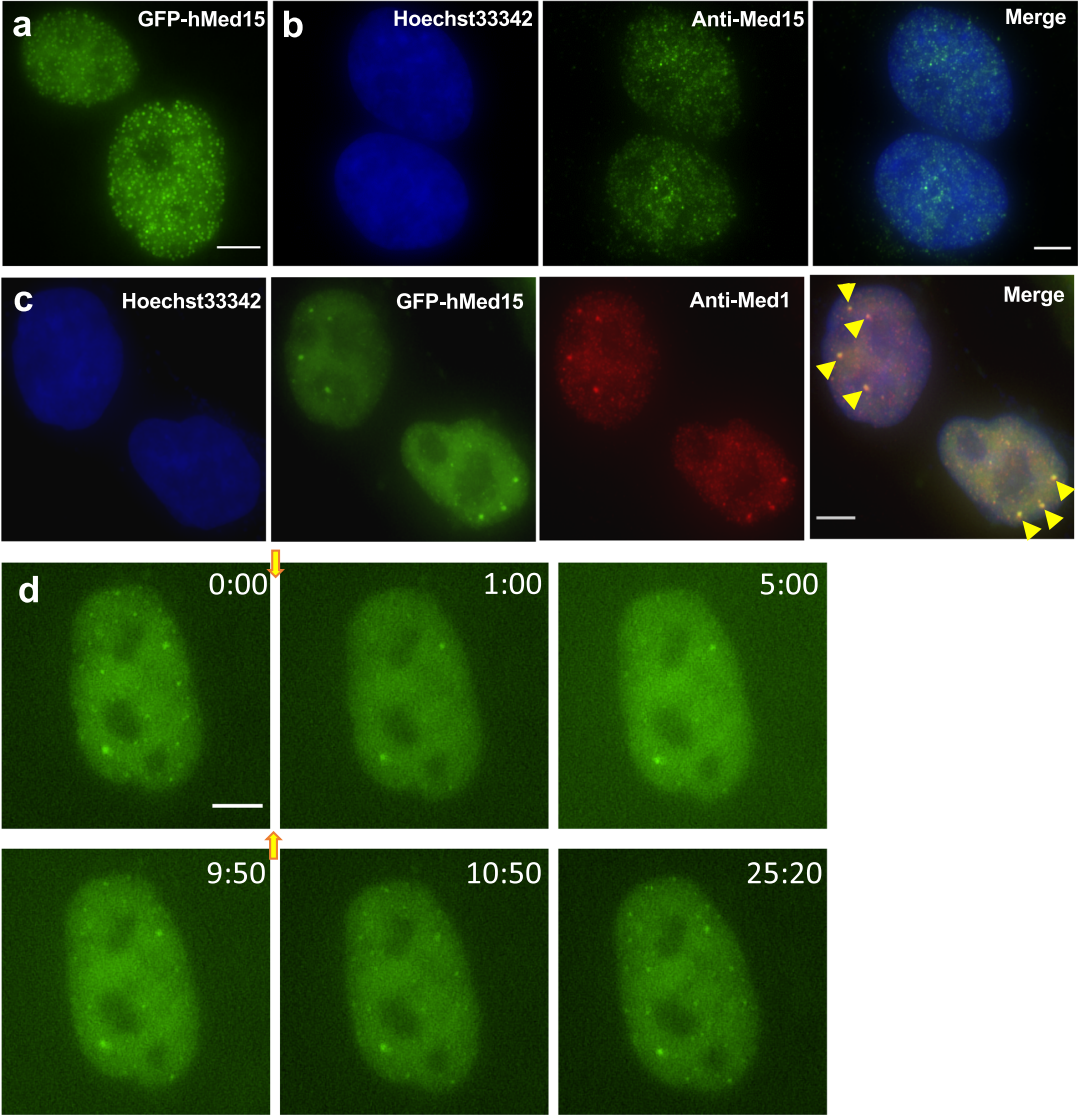
Med15 forms nuclear foci that are disrupted by Hexanediol treatment. **a** Fluorescence image of a U2OS cell transfected with AcGFP-hMed15. **b** Fluorescence images of U2OS cells stained with Hoechst33342 (blue) and an anti-Med15 antibody (green). Similar results were obtained from three independent experiments. **c** Fluorescence images of a human T24 cell line stably expressing GFP-hMed15 (green) and co-stained with an anti-Med1 antibody (red) and Hoechst33342 (blue). Yellow arrowheads indicate sites of colocalization. Similar results were obtained from three independent experiments. **d** Time-lapse fluorescence images of a cell from human T24 stable cell line expressing AcGFP-hMed15 during treatment with 0.5% 1, 6-Hexanediol and subsequent recovery. Yellow arrows indicate the time points of Hexanediol addition and withdrawal. Noted time points on the images are in mm:ss format. Similar results were obtained from three independent experiments. All scale bars: 5 μm

The Mediator complex consists of over 30 protein subunits, many of which contain IDR sequences [26] that might contribute to phase separation. Thus, it would be interesting to study whether additional Mediator subunits might participate in the formation of nuclear condensates. In this study, we focused on a single subunit, Med15, which contains a large IDR including multiple Glutamine (Q) residues (Additional file 1: Fig. S2a). First, we found that GFP-tagged human Med15 or RFP-tagged mouse Med15 formed multiple nuclear foci in U2OS cells, respectively (Fig. 2a, Additional file 1: Fig. S2b). Consistently, immunofluorescence staining using a Med15 antibody revealed numerous nuclear foci in U2OS cells (Fig. 2b), and Med15 foci detected by immunofluorescence were colocalized with TagRFP-mMed15 (Additional file 1: Fig. S2b). We performed western blot to confirm the specificity of this antibody in detecting endogenous Med15 proteins in U2OS cells (Additional file 1: Fig. S2c). To further characterize the properties of Med15 nuclear condensates, we generated a T24 stable cell line expressing GFP-hMed15. GFP-tagged human Med15 formed multiple nuclear foci (Additional file 1: Fig. S2d) and were colocalized with Med15 foci detected by immunofluorescence (Additional file 1: Fig. S2e). Furthermore, we found that all prominent GFP-hMed15 foci were colocalized with nuclear foci formed by endogenous Med1 in this stable cell line (Fig. 2c). Therefore, we concluded that both endogenous and overexpressed Med15 formed nuclear condensates in human cells.

We attempted to determine the state of Med15 foci in mitotic cells but did not obtain conclusive results. Med15 immunofluorescence staining revealed multiple foci in mitotic U2OS cells (Additional file 1: Fig. S3a) and Med15 foci numbers per cell were higher in mitotic cells than in interphase cells (Additional file 1: Fig. S3b). In the stable T24 cell line expressing GFP-Med15, however, prominent Med15 foci observed in interphase cells were absent in most mitotic cells (Additional file 1: Fig. S3c, d). We suggest that prominent GFP-Med15 foci in the stable cell line that were colocalized with anti-Med1 (Fig. 2c) may be more consistent markers of Mediator condensates reported in previous studies [13, 14].

Next, we examined the state of Mediator condensates in U2OS cells where Med15 was depleted by RNAi. Both Med15 and Med1 protein levels appeared to be reduced in Med15 knockdown cells (Additional file 1: Fig. S4c), and Med15 mRNA level was substantially lower (Additional file 1: Fig. S4d). Anti-Med15 staining was reduced to background levels in Med15 knockdown cells (Additional file 1: Fig. S4a), confirming the specificity of this antibody in immunostaining experiments. Notably, anti-Med1 staining intensity was diminished and the numbers of Med1 nuclear foci were significantly decreased in Med15 knockdown cells (Additional file 1: Fig. S4a, b). Our results thus suggested Med15 was important for both maintaining Med1 protein level and forming Med1 nuclear foci. Additionally, GFP-hMed15 expressed in Med15 knockdown cells formed nuclear foci similarly as in control cells (Additional file 1: Fig. S4e).

Furthermore, we examined the response of GFP-Med15 nuclear foci to Hexanediol treatment by live cell imaging. Because high concentrations of Hexanediol likely introduce non-specific effects to cells [27], we tested Hexanediol concentrations lower than previously used to examine nuclear condensates in the GFP-Med15 stable cell line. Interestingly, application of 0.5% 1, 6-Hexanediol resulted in rapid and substantial decrease of fluorescence intensities of GFP-Med15 nuclear foci (Fig. 2d, Additional file 1: Fig. S5, Additional file 2: Video S1), and withdrawing Hexanediol from the growth media resulted in the reassembly of GFP-Med15 foci that plateaued in about 15 min (Fig. 2d, Additional file 1: Fig. S5, Additional file 2: Video S1). Therefore, rapid disruption/reassembly upon 1,6-Hexanediol treatment/withdrawal is a property shared between Med15 foci and Med1 foci. Our results indicated that low concentrations of Hexanediol (i.e., 0.5%) could dissolve nuclear condensates that have small sizes (such as GFP-Med15 foci).

### Dynamics of Med15 foci in living cells

**Fig. 3:**
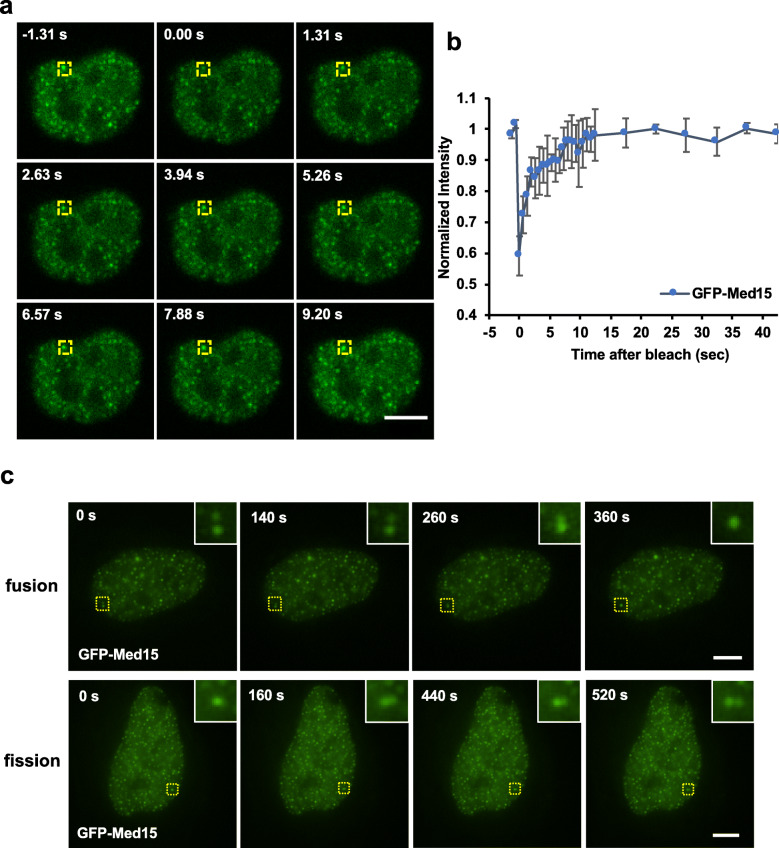
Dynamics of Med15 nuclear foci in living cells. **a** Time-lapse images of an NIH3T3 cell nucleus expressing AcGFP-mMed15 during a FRAP experiment. Yellow boxes indicate the photobleached area. *t* = 0.00 s indicates the time point immediately after photobleaching. **b** Plot of GFP-mMed15 fluorescence intensity at the photobleached area within 42 s after photobleaching. Time intervals between individual frames in the first 20 cycles and the last 20 cycles of post-bleach were 655 ms and 5 s, respectively. Data are presented as the mean ± SEM, *n* = 3. **c** Time-lapse images of GFP-mMed15 foci that exhibited fusion and fission events (highlighted in yellow boxes and enlarged at upper-right insets). All scale bars: 5 μm

A characteristic feature of liquid-like nuclear condensates is the rapid exchange of their molecular components with the nucleoplasm [13, 14]. We next examined the association of Med15 with nuclear foci in living cells by fluorescence recovery after photobleaching (FRAP). In NIH3T3 cells expressing AcGFP-Med15, we observed that fluorescence intensities of nuclear foci recovered to approximately initial levels within 10 s after initial photobleaching (Fig. 3a, b). Similar results were obtained from NIH3T3 cells expressing TagRFP-Med15 (Additional file 1: Fig. S6). Furthermore, we observed fusion events and fission events of GFP-Med15 foci on the timescale of several minutes (Fig. 3c, Additional file 1: Fig. S7, Additional file 3: Video S2, Additional file 4: Video S3). Therefore, our results indicated that Med15 exchanged between nuclear condensates and the nucleoplasm at a rate comparable with that measured on Med1 [13], and suggested that Med15 molecules within these nuclear condensates were in a liquid-like phase.

### DYRK3 overexpression disrupts Med1 nuclear foci and Med15 nuclear foci.

Recent studies revealed that the dual-specificity tyrosine kinase DYRK3 played a key role in dissolving multiple, but not all membraneless organelles during mitosis [9]. Moreover, overexpressing DYRK3 disrupted several nuclear organelles (such as nuclear speckle and Cajal body) in interphase cells [9]. We hypothesized that DYRK3 might play a role in the dissolution of Med1 foci during mitosis. As expected, Med1 foci were mostly dissolved in cells synchronized at mitotic stage by thymidine-nocodazole block (Additional file 1: Fig. S8a). Notably, Med1 foci reappeared in a portion of mitotic cells upon treatment with GSK626616, a small molecule inhibitor of DYRK3 (Additional file 1: Fig. S8b-d), suggesting that DYRK3 kinase activity plays a role in dissolving Med1 foci in mitotic cells.

**Fig. 4:**
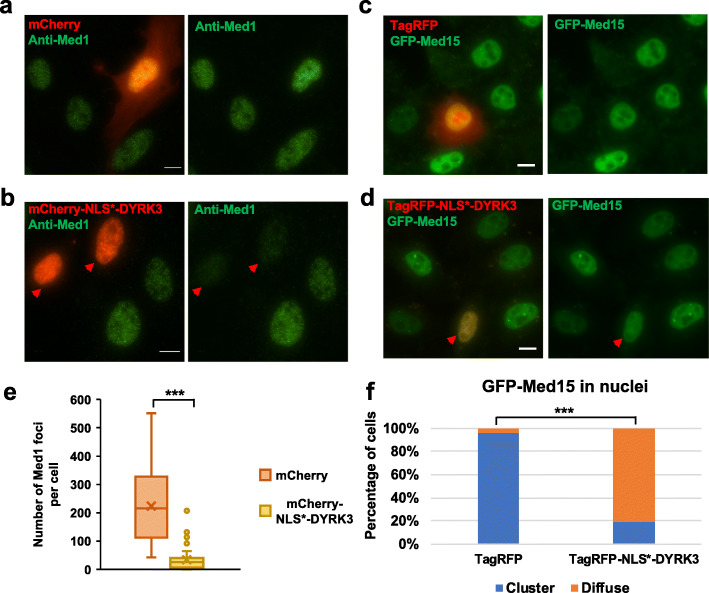
DYRK3 overexpression disrupts Med1 foci and Med15 foci. **a, b** Fluorescence images of U2OS cells transfected with mCherry (**a**) or mCherry-NLS*-DYRK3 (**b**) and stained with anti-Med1 (green). **c, d** Fluorescence images of human T24 cells that stably expressed GFP-Med15 (green) and were transfected with TagRFP (**c**) or TagRFP-NLS*-DYRK3 (**d**). All scale bars are 10 μm. In **b** and **d**, red arrowheads indicate the transfected cells. Similar results were obtained from three independent experiments. **e** The number of Med1 clusters quantified in individual U2OS cells after transfection with mCherry (*n* = 29) or mCherry-NLS*-DYRK3 (*n* = 44) (Intensity threshold: 600). Student’s *t* test: *p* < 0.001 (indicated by ***). **f** The percentage of cells displaying clusters of GFP-Med15 or diffuse localizations in the nucleoplasm after transfection with TagRFP (*n* = 28) or TagRFP-NLS*-DYRK3 (*n* = 57). Fisher’s exact test: *p* < 0.001 (indicated by ***)

Next, we examined the effects of DYRK3 overexpression on Med1 and Med15 nuclear foci in interphase cells. We expressed mCherry or mCherry-NLS*-DYRK3 (NLS*: SV40 nuclear localization signal) in U2OS cells and performed immunofluorescence staining against Med1. Most cells overexpressing DYRK3 showed diffuse Med1 localization in the nucleoplasm, in which the numbers of Med1 nuclear foci were substantially decreased (Fig. 4a, b, e). The same results were obtained in NIH3T3 cells (Additional file 1: Fig. S9). In the T24 cell line stably expressing GFP-Med15, most cells transfected with TagRFP-NLS*-DYRK3 lost prominent GFP-Med15 foci that were observed in untransfected interphase cells (Fig. 4c, d, f). Interestingly, dissolution of GFP-Med15 foci was affected by relative expression levels of TagRFP-NLS*-DYRK3. The mean intensity of TagRFP-NLS*-DYRK3 in lentivirus-infected cells was ~ 7 fold lower than that in transfected cells (Additional file 1: Fig. S10b), and GFP-Med15 foci were still present in most lentivirus-infected cells (Additional file 1: Fig. S10a, c). Likewise, transfected cells containing GFP-Med15 foci had significantly lower mean intensity of TagRFP-NLS*-DYRK3 compared to those without visible GFP-Med15 foci (Additional file 1: Fig. S10d). Taken together, our work revealed that Med1 foci and GFP-Med15 foci can be dissolved by overexpressing DYRK3 kinase, which provides a likely explanation for the dissolution of Med1 foci and GFP-Med15 foci in mitotic cells.

Because the Serine-rich IDR region of Med1 was shown to mediate its phase separation in vitro [13], we tested whether overexpressing DYRK3 could affect nuclear condensates formed by Med1 IDR in cells. Interestingly, when GFP-tagged Med1 IDR region (amino acid residues 948-1568) was expressed in NIH3T3 cells, it was enriched in the nucleolar regions and colocalized with Nucleophosmin (NPM1), an abundant nucleolar protein (Additional file 1: Fig. S11a). Notably, expressing TagRFP-NLS*-DYRK3 resulted in the redistribution of GFP-Med1 (948-1568) to the nucleoplasm (Additional file 1: Fig. S11b).

### The Q-rich IDR and a hydrophobic amino acid region of Med15 are both required to form nuclear condensates

**Fig. 5:**
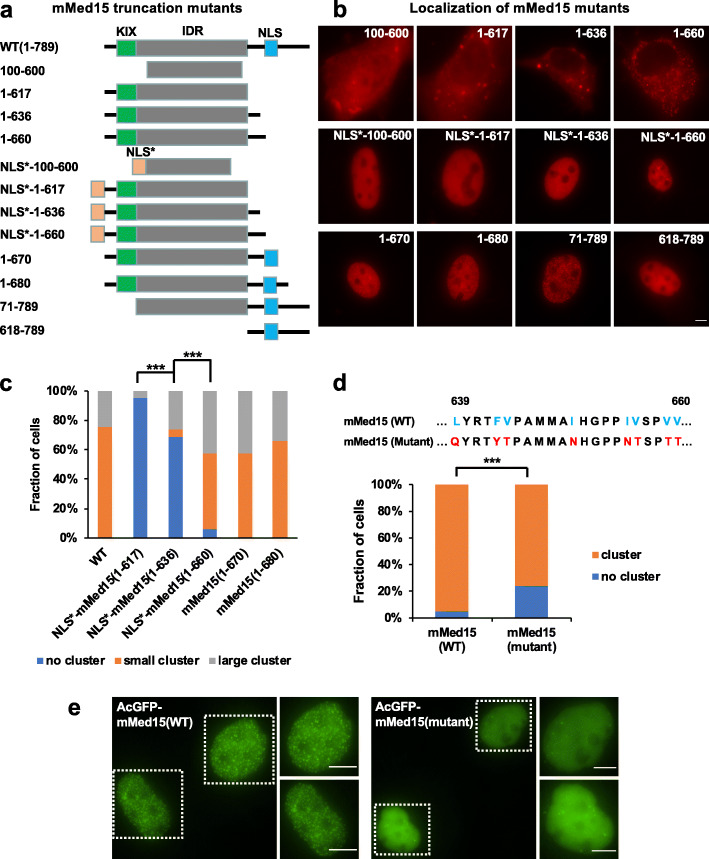
Formation of Med15 nuclear foci is mediated by its IDR and a hydrophobic amino acid sequence. **a** Diagrams of mouse Med15 truncation mutants examined in this study. TagRFP was fused to the N-terminus of each protein fragment. SV40 NLS (NLS*, orange) was inserted before the coding regions of several Med15 truncation mutants at their N-termini. The endogenous NLS (blue) of mouse Med15 is located at amino acid residues 661-670. **b** Representative images of NIH3T3 cells expressing each mouse Med15 truncation mutant fused to TagRFP. Scale bar: 5 μm. **c** Percentages of NIH3T3 cells that display no nuclear clusters, small nuclear clusters (diameter < 1 μm), and large nuclear clusters (diameter > 1 μm) of mouse Med15 (WT) or truncation mutants. The numbers of analyzed cells were 90, 88, 96, 117, 92, and 85, respectively. The numbers of cells without clusters and the numbers of cells with clusters (including small and large) were obtained for each construct and subject to Fisher’s exact test: *** indicates *p* < 0.001. **d** Percentages of cells that display no clusters or nuclear clusters among NIH3T3 cells expressing GFP-mMed15 (WT) (*n* = 73) or GFP-mMed15 (mutant) (*n* = 164). This Med15 mutant contains eight point mutations within the 639-660 region which convert hydrophobic amino acids into hydrophilic amino acids (shown in the sequence comparison above the plot). Fisher’s exact test: *p* < 0.001 (indicated by ***). **e** Representative images of NIH3T3 cells expressing AcGFP-tagged mMed15 (WT) protein and the Med15 mutant described in **d**. The right columns contain the enlarged images of cells marked with dashed white borders. Scale bars: 5 μm. Similar results were obtained from two independent experiments

Next, we sought to identify the amino acid regions responsible for the formation of Med15 nuclear condensates. We generated a series of mouse Med15 truncation mutants fused to TagRFP at its C-terminus and compared their abilities to form nuclear foci (Fig. 5a). Med15 contains a KIX domain at its N-terminus, followed by a long glutamine-rich IDR (71-617) and a structured C-terminal domain that also contains its NLS (661-670). Surprisingly, Med15 (100-600) and Med15 (1-617) fragment fused to TagRFP and SV40 NLS (NLS*) were diffusely localized in the nucleus (Fig. 5b, c). Med15 (100-600) and Med15 (1-617) fused to TagRFP only were localized in the cytoplasm and formed several large aggregates (Fig. 5b) distinct from numerous small nuclear foci formed by full-length Med15 (Fig. 2a, Fig. 3c). These results suggested that the glutamine-rich IDR of Med15 was not sufficient to form condensates in the nucleus. These observations were also consistent with previous findings on several prion-like RNA-binding proteins that formed condensates in the cytoplasm while remained soluble in the nucleus [28].

Interestingly, both Med15 (1-670) and Med15 (1-680) formed multiple small nuclear foci (Fig. 5b, c) resembling those formed by full-length Med15. Because  the 661-670 amino acid region is the native NLS of Med15, we examined TagRFP-NLS*-Med15 (1-660) and found that it also formed multiple nuclear foci (Fig. 5b, c). Importantly, both TagRFP-NLS*-Med15(1-660) and TagRFP-Med15(1-680) were colocalized with GFP-Med15 foci (Additional file 1: Fig. S12a). Thus, Med15 (618-660) region likely plays a role in condensate formation. Meanwhile, a C-terminal truncation of Med15 (amino acid 618-789) failed to form nuclear foci (Fig. 5b), suggesting that the N-terminal region (1-617) containing the Q-rich IDR also contributed to nuclear condensate assembly. Furthermore, we generated human Med15 truncation mutants according to the alignment between human and mouse Med15 protein sequences and observed a strong effect of the 616-659 amino acid region in condensate assembly in both wild-type cells (Additional file 1: Fig. S13a, b) and in Med15 knockdown cells (Additional file 1: Fig. S13c, d). Therefore, the mechanisms underlying nuclear condensate formation are likely conserved between mouse and human Med15 proteins.

We next sought to identify the motifs within this region that contribute to the formation of Med15 nuclear condensates. We noticed that mouse Med15 (637-660) region contained eight hydrophobic amino acid residues (Fig. 5d), raising the possibility that hydrophobic interactions may in part mediate the formation of Med15 nuclear condensates. Seven out of the eight hydrophobic amino acid residues are conserved in human Med15. To test this hypothesis, we mutated all eight hydrophobic amino acids in AcGFP-mMed15 to their hydrophilic mimics and found that the mutated protein formed visibly fewer nuclear foci than wild-type Med15 and that a lower fraction of cells showed Med15 foci (Fig. 5d, e). Taken together, although either the glutamine-rich IDR or the hydrophobic amino acid region (637-660) of Med15 was insufficient to form nuclear condensates, synergistic functions from both regions likely resulted in condensate formation.

### Both IDR and C-terminal domain of Med15 contribute to phase separation in optodroplet assays

**Fig. 6:**
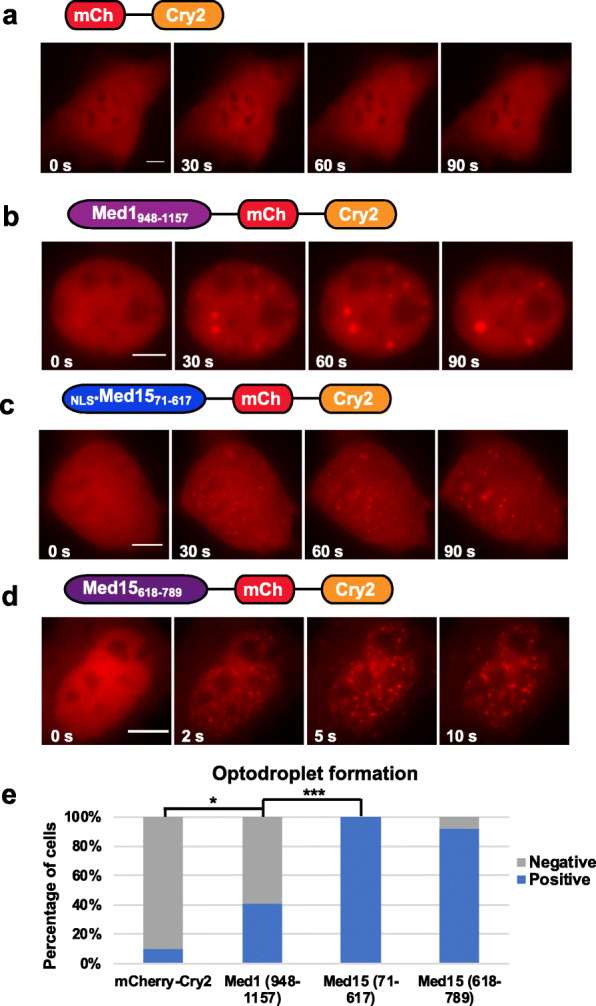
Med1 and Med15 regions induce the formation of optodroplets upon illumination by blue light. **a–d** Representative images during optodroplet activation in NIH3T3 cells expressing the following constructs: mCherrry-Cry2 (**a**), Med1_(948-1157)_-mCherry-Cry2 (**b**), NLS*-Med15_(71-617)_-mCherry-Cry2 (**c**), and Med15_(618-789)_-mCherry-Cry2 (**d**). *t* = 0 s indicates the starting point of blue light illumination. Time intervals between illuminating blue light and image acquisition are noted on each image. All scale bars are 5 μm. Similar results were obtained from three independent experiments. **e** Percentage of cells forming optodroplets after 30 s blue light stimulation at the same intensity. Numbers of cells observed were the following: mCherry-Cry2: 19; Med1_(948-1157)_: 42; Med15_(1-617)_: 18; Med15 _(618-789)_: 26. * and *** indicates *p* < 0.05 and *p* < 0.001 in Fisher’s exact test, respectively

Determining the capacity of Med15 IDR or Med15 C-terminal region (618-789) in promoting phase separation in vivo would benefit from a cellular assay that can visualize condensate formation in real time. We applied the optodroplet assay to analyze how Med15 IDR, Med15 C-terminal domain, or Med1 IDR contribute to phase separation in cells. In this assay, protein domains of interest were fused to a fluorescent protein and the coding sequence of cryptochrome2 (Cry2), a blue light-sensitive protein from *Arabidopsis thaliana*, and the formation of optodroplets after blue light stimulation was visualized in real time [29]. First, we transiently expressed mCherry-Cry2 in NIH3T3 cells and did not observe optodroplet formation after illumination with blue light for 90 s (Fig. 6a). In contrast, mCherry-Cry2 fused to a Serine-rich IDR region of Med1 (amino acid 948-1157) formed optodroplets within 30 s of blue light stimulation (Fig. 6b), consistent with a previous study [13]. Next, we generated constructs of mCherry-Cry2 fused to NLS*-Med15 IDR (amino acid 71-617) or Med15 C-terminal region (amino acid 618-789) and examined optodroplet formation in living cells. Optodroplets formed by Med15 IDR appeared in spherical shape but were smaller in size than those formed by Med1 IDR after the same duration of blue light stimulation (Fig. 6b, c). Remarkably, Med15 C-terminal region formed optodroplets within 5 s after blue light stimulation (Fig. 6d), considerably faster than Med1 or Med15 IDR. The apparently lower efficiency of Med1 IDR in optodroplet formation (Fig. 6e) could arise from the shorter length of Med1 IDR or from our experimental conditions. Therefore, the optodroplet assay confirmed that both Med15 IDR and Med15 C-terminal region contributed to phase separation in cells.

FRAP revealed that optodroplets formed by Med1 IDR or Med15 IDR rapidly recovered with *t*_1/2_ < 10 s, and about 80% recovery was reached at 60 s after photobleaching (Additional file 1: Fig. S14a, b, d), consistent with measurement on optodroplets formed by Med1 IDR in a previous study [13]. In contrast, only about 20% FRAP recovery was observed on optodroplets formed by Med15 C-terminal region at 60 s after photobleaching (Additional file 1: Fig. S14c, d). Thus, Med15 C-terminal domain appeared to drive phase separation more efficiently than Med15 IDR or Med1 IDR in the optodroplet assay and might provide a strong adhesive force for maintaining the Mediator condensates.

### Testing the effects of Hexanediol treatment in transcriptional activation of immediate early genes (IEGs) during the serum response.

**Fig. 7:**
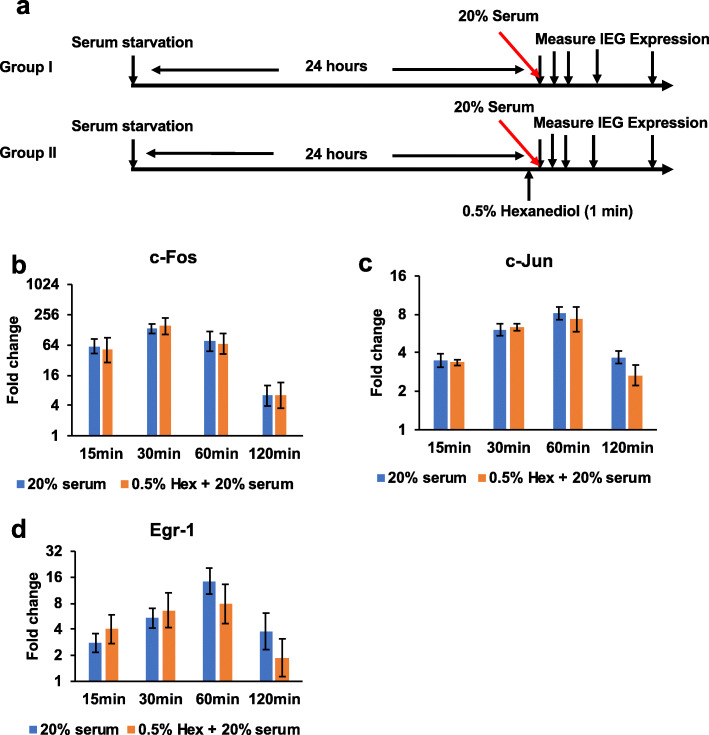
Testing the effects of Hexanediol treatment on IEG activation during serum response. **a** The experimental diagram. In Group I, NIH3T3 cells were serum starved for 24 h and then stimulated with growth media containing 20% serum. In Group II, cells were serum starved for 24 h, treated with 0.5% 1,6-Hexanediol for 1 min and then stimulated with media containing 20% serum but no Hexanediol. IEG expression was measured before adding serum (Group I), before and after Hexanediol treatment (Group II) and at distinct time points after adding 20% serum (Group I & II) by RT-qPCR. **b–d** Fold change of *c-Fos* (**b**), *c-Jun* (**c**), and *Egr-1* (**d**) mRNA expression after serum stimulation in Group I cells (blue bars) and in 0.5% Hexanediol-treated cells (orange bars). Three experimental replicates were performed. In **b**–**g**, data are presented as mean ± SEM, and *y*-axes are plotted in log2 scale to facilitate comparison between time points. *GAPDH* mRNA expression was used for internal controls

Although recent studies have revealed phase separation phenomena of multiple key components of transcriptional machineries [6, 11, 15], roles of these nuclear condensates in transcriptional regulation were less well understood. We explored the roles of nuclear condensates during rapid gene activation by examining the effects of Hexanediol treatment and withdrawal on IEG expression during the serum response. IEGs respond very rapidly to a variety of cell-extrinsic and cell-intrinsic signals, including serum, growth factors, cytokines, and UV radiation [30, 31]. Given that Hexanediol treatment at high concentrations leads to inhibition of kinase and phosphatase activities [27], we compared the effects of 0.5% and 10% Hexanediol on IEG activation. We examined a few well-characterized IEGs (*c-Fos*, *c-Jun*, and *Egr-1*) in this study. NIH3T3 cells were analyzed in two groups. In Group I, cells were serum starved for 24 h and treated with media containing 20% serum. In Group II, cells were serum starved for 24 h, treated with 0.5% or 10% Hexanediol diluted in serum starvation media for 1 min and then stimulated with media containing 20% serum but no Hexanediol. IEG expression at distinct time points was analyzed by RT-qPCR (Fig. 7a, Additional file 1: Fig. S17a). By immunostaining, we found that Med1 and Med15 nuclear foci were both abolished after 1 min treatment with 10% Hexanediol and were restored to pre-treatment levels after 30 min serum induction (Additional file 1: Fig. S15). In a T24 cell line stably expressing GFP-Med15, Med15 foci were rapidly diminished upon 0.5% Hexanediol treatment and restored upon serum induction/Hexanediol withdrawal (Additional file 1: Fig. S16, Additional file 5: Video S4).

We found that transcriptional activation of *c-Fos*, *c-Jun*, and *Egr-1* genes was significantly delayed in cells pretreated with 10% Hexanediol but minimally affected in cells pretreated with 0.5% Hexanediol. Highest levels of IEG expression were found at about 30 min after serum induction in Group I cells but instead at 60 min or 120 min after serum induction in cells pretreated with 10% Hexanediol (Additional file 1: Fig. S17b-d). However, disruption of Mediator condensates by 0.5% Hexanediol prior to serum induction (Additional file 1: Fig. S16, Additional file 5: Supplementary Video S4) did not result in a delay in IEG expression (Fig. 7b–d). Whether the presence of 0.5% Hexanediol during serum stimulation can affect IEG activation remains to be tested. Ideally, molecular reagents with improved specificity would help to better understand the functions of Mediator condensates in inducible gene expression.

Importantly, Med15 knockdown attenuated IEG activation. We found that expression of c*-Fos* and *Egr-1* in Med15 knockdown cells after 30 min serum induction was 2–3 fold lower than wild-type U2OS cells (Additional file 1: Fig. S18a, c). Most substantial decrease in expression levels of all three IEGs upon Med15 knockdown was found at 60 min serum induction (Additional file 1: Fig. S18a-c). Thus, Med15 knockdown impairs the functions of the Mediator complex in regulating IEG activation upon serum induction.

## Discussion

**Fig. 8:**
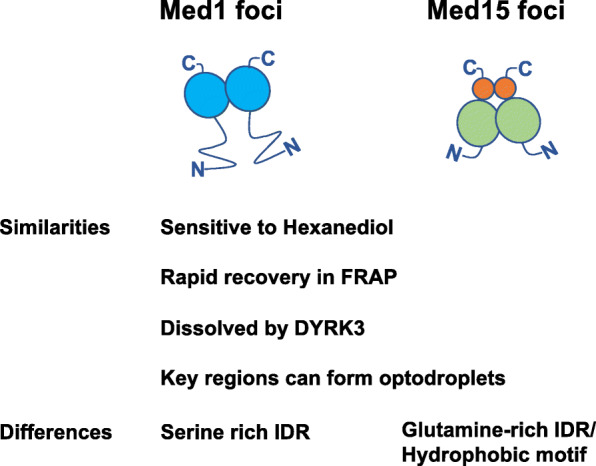
Characteristics of Med1 foci and Med15 foci. Formation of Med1 foci is mediated by the serine-rich IDR (blue circle). Formation by Med15 foci requires both the glutamine-rich IDR (green circle) and a short hydrophobic motif (orange dot). Shared and distinct features between Med1 foci and Med15 foci are described

Our study revealed several common features of nuclear condensates formed by Med1 and Med15 (Fig. 8). Nuclear condensates formed by Med1 and Med15 were dissolved upon treatment with 1,6-Hexanediol and reassembled upon withdrawal (Fig. 2d, Additional file 1: Fig. S1, S5, S15, S16). Both Med1 foci [13] and Med15 foci showed a rapid FRAP recovery (Fig. 3a, b). Like many nuclear organelles, Med1 foci and GFP-Med15 foci were dissolved in mitotic cells (Fig. 1b, c, Additional file 1: Fig. S3c, d) and presumably reassembled as cells exit mitosis. This was consistent with the notion that intracellular organelles were dissolved in mitosis by increased DYRK3 kinase activities and with the findings that DYRK3 overexpression dissolved some nuclear organelles [9], Med1 foci and GFP-Med15 foci in interphase cells (Fig. 4, Additional file 1: Fig. S9). We also found that dissolution of GFP-Med15 foci was affected by the expression levels of TagRFP-NLS*-DYRK3 (Fig. 4d, Additional file 1: Fig. S10). Additional insights may be obtained by measuring subcellular concentrations of Mediator subunits and DYRK3.

Our study revealed interesting new insights on nuclear condensate formation. The Q-rich IDR of Med15 was unable to form condensates when expressed in the nucleus alone, while a short hydrophobic motif was required to assist in condensate assembly (Fig. 5). This finding was consistent with the notion that hydrophobic residues could serve as adhesive elements in phase-separating IDR and promote condensate formation [3]. Moreover, C-terminal region of Med15 could rapidly form optodroplets, which had a much slower FRAP recovery compared with optodroplets formed by Med15 or Med1 IDRs (Additional file 1: Fig. S14a-d) and appeared as irregular shapes in some cases (Additional file 1: Fig. S14e), suggesting that Med15 C-terminal domain could drive formation of condensates “deep” in the phase diagram that could transition from a fully mobile liquid-like state into a less mobile gel-like state [29].

Both Med1 and Med15 foci exhibited features shared by many biomolecular condensates, such as sensitivity to 1,6-Hexanediol and rapid FRAP recovery. Nonetheless, these are not definitive diagnostics that a cellular structure was formed by LLPS [3]. As a thermodynamic principle, phase separation is exhibited by unmixing of components due to biomolecular interactions within two distinct phases that outweigh the increase in entropy by mixing the two phases and result in a lower free energy state [1]. Modulating interaction modules or protein concentrations clearly altered phase separation outcomes in vitro [32]. Consistent with these principles, interactions within the Mediator complex, between Mediator subunits, or between Mediator and TFs might lead to phase separation. Nonetheless, formation of Mediator condensates does not necessarily exclude affinity-based macromolecular assembly as an alternative explanation. It is of interest to note that DNA-mediated compartmentalization distinct from LLPS occurred during viral infection [33]. As recently discussed [34], the roles of LLPS vs other biochemical processes in nuclear condensate formation would need to be further studied.

The tail module of the Mediator complex interacts with multiple transcription activators and participates in various signal-induced gene expression programs [16, 19]. As expected, Med15 knockdown by RNAi substantially reduced IEG activation during the serum response (Additional file 1: Fig. S18). Med15 knockdown reduced Med15 and Med1 protein levels (Additional file 1: Fig. S4a, c) and abolished both Med15 foci and Med1 foci (Additional file 1: Fig. S4a, b). Surprisingly, Med15 C-terminal domain exhibited higher efficiency than Med15 IDR or Med1 IDR in promoting phase separation in the optodroplet assay (Fig. 6, Additional file 1: Fig. S14). Thus, Med15 exhibits several interesting features and might provide clues for better understanding the formation and regulation of transcription coactivator condensates in mammalian cells.

Formation of nuclear condensates provides potential explanations for interactions between Mediators and activation domains of multiple TFs [15] or between RNA Pol II C-terminal domain and splicing machineries [12]. These microenvironments formed within the cell nucleus were thought to facilitate cooperative interactions between transcription components and might enable rapid gene activation upon environmental signaling [35]. We explored the roles of Mediator condensates in rapid IEG activation by treating cells with Hexanediol prior to serum stimulation. The effects of Hexanediol on IEG activation during the serum response were concentration-dependent and could not be unambiguously associated with Mediator condensates (Fig. 7, Additional file 1: Fig. S17). For better understanding the roles of Mediator condensates in gene expression, it would be helpful to develop new perturbation approaches and to examine genomic regions closely associated with these condensates.

## Conclusions

Understanding the formation, regulation and functions of nuclear condensates has become an exciting research field. We have revealed the Mediator complex subunit Med15 formed nuclear condensates in mammalian cells and characterized their features using multiple imaging-based approaches. Med15 condensates shared several features with Med1 condensates, such as sensitivity to Hexanediol, rapid FRAP recovery, and dissolution by DYRK3. Interestingly, the formation of Med15 condensates requires not only the glutamine-rich IDR but also a hydrophobic amino acid motif. Both IDR and C-terminal region of Med15 contributed to phase separation in the optodroplet assay. Our work has therefore reported multiple novel features of Med15 nuclear condensates and identified a bipartite formation mechanism.

## Methods

### Cell culture and transfection

NIH3T3 cells and U2OS cells were obtained from Chinese Academy of Sciences, Kunming Cell Bank (www.kmcellbank.com). T24 (ATCC HTB-4) human urinary bladder cancer cells were kindly provided by Prof. Tiebang Kang (Sun Yat-sen University Cancer Center). HEK 293 T cells were obtained from ATCC.

NIH3T3 cells were cultured in high-glucose Dulbecco’s modified Eagle’s medium (DMEM, Thermo Fisher, C11995500) supplemented with 10% newborn calf serum (NCS, Thermo Fisher, 16010-159) and 100 U/ml penicillin-streptomycin (Thermo Fisher, 15140-122) at 37 °C with 5% CO_2_ in a humidified incubator. U2OS cells were cultured in low glucose Dulbecco’s modified Eagle’s medium (DMEM, Thermo Fisher, C11885500) supplemented with 10% fetal bovine serum (FBS, Hyclone), 1× GlutaMAX supplement (Thermo Fisher, 35050-061), 100 U/ml penicillin-streptomycin at 37 °C with 5% CO_2_ in a humidified incubator. T24 cells were cultured in RPMI-1640 medium (Gibco, C11875500BT) supplemented with 10% FBS (AusgeneX, FBSSA500-S) and 100 U/ml penicillin-streptomycin at 37 °C with 5% CO_2_ in a humidified incubator. HEK 293T cells were cultured in high-glucose DMEM (Gibco, C11995500BT) supplemented with 10% FBS (AusgeneX, FBSSA500-S). Transfection was performed using Lipofectamine 3000 Transfection Kit (Thermo Fisher, L3000-015) following the manufacturer’s instructions.

### Western blot

U2OS cells were lysed in cold lysis buffer (150 mM NaCl, 1% NP-40, 0.5% Sodium deoxycholate, 0.1% SDS, 25 mM Tris, and 1 mM PMSF). Cell lysates were then prepared by a sonicator (Fisher Scientific, FB120) at 35% power for 1 min and cleared by centrifugation at 12,000*g* for 20 min. 1/4 volume of SDS-PAGE loading buffer was added to the supernatant and boiled for 10 min in a dry thermostat. Cell lysates were separated on an 8% polyacrylamide gel by SDS-PAGE and transferred to a nitrocellulose membrane. The membrane was then blocked with 5% non-fat milk and incubated with an anti-TRAP220/MED1 antibody (Abcam, ab64965) at 1:1000 dilution or an anti-PCQAP/Med15 antibody (Abcam, ab181158) at 1:1500 dilution overnight at 4 °C. The membrane was then incubated with a goat anti-rabbit IgG (H + L) horseradish peroxidase secondary antibody (Invitrogen, A16110) diluted by 1:10,000 in 1× PBST with 5% non-fat milk at room temperature for 1 h. Chemiluminescence signals were detected by SuperSignal West Pico PLUS Chemiluminescent Substrate (Thermo Fisher, 34577) and visualized on a Tanon-5200 Chemiluminescence Imaging System (Tanon Science and Technology, Shanghai, China). Original western blot images are provided in Additional file 1: Fig. S19.

### Immunofluorescence staining

NIH3T3 and U2OS cells were cultured as described above. Prior to immunostaining experiments, cells were plated on Lab-Tek CC2 chamber slides (Thermo Fisher, 154852) at approximately 50% confluency. 12–20 h after plating or after transfection, cells were subject to treatment and fixed for 15 min at room temperature using 4% paraformaldehyde (Thermo Fisher, 28908) in 1× PBS. Fixed cells were washed with 1× PBS, permeabilized with 0.05% Triton X-100 in PBST (1× PBS + 0.05% Tween-20) for 10 min at room temperature, washed with 1× PBS again, and blocked with 2% BSA (Sigma, B2064) in PBST. Cells were then incubated with an anti-TRAP220/MED1 antibody (Abcam, ab64965) diluted by 1:1000, an anti-GFP antibody (Cell Signaling Technology, 2956) diluted by 1:500, or an anti-PCQAP/MED15 antibody (Abcam, ab181158) diluted by 1:75 in PBST with 2% BSA at 4 °C overnight. The next day, cells were washed three times with PBST for 30 min at room temperature and were then incubated with Alexa Fluor 488 goat anti-rabbit IgG(H+L) (Invitrogen, A11008) diluted by 1:1000 in PBST with 2% BSA. Cells were then washed three times with PBST for 30 min at room temperature and stained with 1 μg/mL Hoechst33342 (Novon Scientific, China, SS0160) for 5 min at room temperature in the dark. Cells were mounted with Vectashield antifade mounting medium (VectorLabs, Burlingame, CA, USA) and slides were stored at a -20 °C freezer until image acquisition. Z-stack images were acquired at a Nikon Eclipse Ti2-E wide-field fluorescence microscope using a 60× oil-immersion objective (numerical aperture 1.4) and a DS-Qi2 CMOS camera. The Z-interval was 0.3–0.5 μm. A 1.5× magnifier lens was placed in the light path during imaging.

### Image and statistical analysis

All images were post-processed using ImageJ (https://imagej.net/Fiji). Changes in fluorescence intensities at Med15 nuclear foci during FRAP were measured by ImageJ. The numbers of nuclear foci per cell were generated by the AirLocalize program in MATLAB (The Mathworks Inc., Natick, MA). For statistical analysis, Fisher’s exact test was performed using GraphPad Prism 9 (https://www.graphpad.com/quickcalcs/contingency1.cfm) and Student’s *t* test was performed using GraphPad Prism 7.0.4. *p* < 0.05 was considered to be statistically significant.

### Molecular cloning

Mouse Med15, human Med15, and human DYRK3 cDNA were amplified by RT-PCR from total RNA extracted from NIH3T3 cells and U2OS cells, respectively. cDNAs were synthesized by RevertAid First Strand cDNA synthesis kit (Thermo Fisher, K1266) and amplified by Phusion DNA polymerase (Thermo Fisher, F530L). Amplified Med15 full-length cDNA and truncated cDNA fragments were then digested by EcoRI and KpnI restriction enzymes and cloned into pAcGFP-C1 or pTagRFP-C vectors. DYRK3 cDNA was linked to the DNA sequence encoding SV40 nuclear localization signal (CCGAAGAAGAAGCGAAAGGTC) at its N-terminus and cloned into pAcGFP-C1, pTagRFP-C, or pmCherry-C1 using EcoRI and BamHI restriction enzymes. The IDR region (amino acid 948-1568) of mouse Med1 cDNA was amplified by RT-PCR and inserted into pAcGFP-C1 vector using EcoRI and ApaI restriction enzymes. Cry2 cDNA was synthesized by Genscript (Nanjing, China). mCherry-Cry2 and Med1_(948-1157)_-mCherry-Cry2 optodroplet constructs were generated by PCR of Cry2 cDNA and cloning into mCherry-C1 vector. NLS*-Med15_(71-617)_-mCherry-Cry2 and Med15_(618-789)_-mCherry-Cry2 optodroplet constructs were generated with ClonExpress MultiS One Step Cloning Kit (Vazyme Biotech, C113) according to the manufacturer’s instructions. Primers used to clone each cDNA and mutants are provided in Additional file 6: Table S1, S2.

The 8-amino acid point mutant of mouse Med15 was generated by Phusion Site-Directed Mutagenesis Kit (Thermo Fisher, F541) following the manufacturer’s instructions. Multiple rounds of mutagenesis were performed to obtain the Med15 mutant with 8 hydrophobic amino acids mutated to hydrophilic ones (Fig. 5d). The resulting colonies were then screened by sequencing to identify the correct mutants.

### Lentivirus production and stable cell line generation

GFP-human Med15 and TagRFP-human DYRK3 fusion protein were subcloned into the lentiviral expression vector pSin-EF2 [36] by ClonExpress II One Step Cloning Kit (Vazyme Biotech, C112). Primers used to clone lentiviral vectors are provided in Additional file 6: Table S3. The human embryonic kidney 293T cell line was used as a host for virus packaging. The recombinant plasmid pSin-EF2-GFP-hMed15 or pSin-EF2-TagRFP-NLS*-DYRK3 was mixed with psPAX2 and pMD2.G plasmids (at a mass ratio of 3: 2: 1) and co-transfected into HEK 293T cells at 50–60% confluency in a 6-well plate using Lipofectamine 3000 Transfection Kit following the manufacturer’s instructions. Lentivirus was harvested 48 h post-transfection and used to transduce T24 cells. Then, 48 h after transduction, T24 cells were selected with RPMI-1640 medium containing 10% fetal bovine serum and 0.5 μg/mL puromycin (InvivoGen, ant-pr-1) for 2 weeks.

### RNAi

Small interfering RNA (siRNA) sequences targeting human Med15 were designed and synthesized by Genepharma Company (Shanghai, china). To obtain a transient Med15 knockdown, U2OS cells were transfected with 200 nmol/L siRNA targeting Med15 for 72 hours using Lipofectamine 3000 Transfection Kit following the manufacturer’s instructions. Sequences for human Med15 siRNA pool were as follows: 5′– CCAAGACCCGGGACGAAUA–3′, 5′–GGGUGUUGUUAGAGCGUCU–3′, 5′–GGUCAGUCAAAUCGAGGAU–3′, and 5′–CCGGACAAGCACUCGGUCA–3′. A non-targeting scrambled siRNA was used as the negative control: 5′–UUCUCCGAACGUGUCACGUTT–3′.

### Live cell imaging

For all live cell imaging experiments, cells were plated onto 35 mm glass bottom dishes (Cellvis, D35-20-1-N). Time-lapse images of each GFP- or TagRFP-fusion protein in NIH3T3 cells were acquired using a Nikon Eclipse Ti2-E Inverted Microscope equipped with a stage top incubator (Tokai Hit model STX) at 37 °C, 5% CO_2_, and humidity control. All live cell images were acquired with a 60× oil-immersion objective (CFI Plan Apochromat Lambda, numerical aperture 1.4) while using the TI2-N-ND-P perfect focus unit (Nikon) to maintain image focus during acquisition. A 1.5× magnifier lens was placed in the light path to obtain the pixel size of 81.4 nm. A 32× neutral density filter was applied after the fluorescence mercury lamp (C-HGFI, Nikon) to attenuate the excitation light. GFP or mCherry/TagRFP fluorescence was collected through a C-FL-C FITC filter cube (MBE44725, Nikon) or a C-FL-C Texas Red filter cube (MBE46105, Nikon), respectively.

### Hexanediol treatment and withdrawal

We prepared a stock solution of 30% 1, 6-Hexanediol dissolved in ultrapure water and filtrated with 0.22 μm microporous membrane. For immunofluorescence staining, the old culture medium was first removed, and cells were washed three times with 1× PBS. We then carefully added 10% Hexanediol (diluted with the old culture media) along the side wall of the dish and immediately placed in a 37 °C incubator for 1 min. Finally, Hexanediol-containing medium was replaced with normal growth medium after gently washing the cells twice with 1× PBS and once with culture medium. Cells were fixed at each described time point and processed for immunostaining. For live cell imaging of Med15 foci, the culture medium was replaced with growth medium containing 0.5% Hexanediol by a custom-made injection device after acquiring baseline images for about 5 min. Cells were then imaged in 0.5% Hexanediol for about 10 min. Hexanediol-containing medium was replaced by normal growth medium and image acquisition was continued until Med15 foci visibly recovered. Time interval between each frame was 10 s and the exposure time was 200 ms. All images were analyzed with ImageJ.

### Fluorescence recovery after photobleaching (FRAP)

NIH3T3 cells were plated on 35 mm glass bottom dishes and transfected with GFP-Med15 or TagRFP-Med15 plasmid for 24 h before imaging. Next, FRAP was performed at a Leica SP8 STED confocal microscope with a 93× glycerol-immersion objective (numerical aperture 1.3). Cells were maintained at 37 °C and 5% CO_2_ in a humidified stage top incubator during experiment. Five iterations of bleaching were performed with a 488 nm laser or a 561 nm laser at 100% laser power and images were collected every 655 ms. Two and four images were acquired before bleaching for GFP-Med15 and TagRFP-Med15, respectively. Forty images were acquired after bleaching, and the time intervals for the first 20 cycles and the last 20 cycles were 655 ms and 5 s, respectively. Imaging settings were as follows: 8-bit image depth, × 4 zoom (122.55 nm pixel size), 256 × 256 frame size. Fluorescence intensities at the bleached locus (*I*_*L*_), unbleached nuclear area (*I*_*N*_) and at area without cells (*I*_*B*_) were measured at each time point using ImageJ. Pre-bleaching fluorescence intensities at the locus *I*_*L*_*(pre)*, unbleached area *I*_*N*_*(pre)*, and area without cells *I*_*B*_*(pre)* were determined by averaging first two image frames. Normalized fluorescence intensities of Med15 foci during FRAP were determined using the following equation:

$$ F(t)=\frac{\left[{I}_L(t)-{I}_B(t)\right]/{I}_L(pre)-{I}_B(pre)\Big]}{\left[{I}_N(t)-{I}_B(t)\right]/\left[{I}_N(pre)-{I}_B(pre)\right]} $$

*F(t)* was measured from multiple cells in each FRAP experiment, and comparable results were obtained from three independent experiments.

### Cell synchronization and DYRK3 inhibition

U2OS cells growing at log phase were plated at approximately 30% confluency. 16 h after plating, Thymidine was added at a final concentration of 2 mM to block the cell cycle for 24 h. Cells were then washed three times with pre-warmed 1× PBS and were replaced with complete growth medium to release the block. After 4 h, Nocodazole (dissolved in DMSO) was added at a final concentration of 25 ng/mL to the medium and cells were incubated for 12 h. 1 μM GSK626616 (dissolved in DMSO) was added to the media at 6 h after starting the Nocodazole block and incubation was continued for 6 h.

### Optodroplet assay

NIH3T3 cells were grown on 35 mm glass bottom dishes and transfected with mCherry-Cry2, Med1_(948-1157)_-mCherry-Cry2, NLS*-Med15_(71-617)_-mCherry-Cry2, and Med15_(618-789)_-mCherry-Cry2 plasmid for 24 h using Lipofectamine 3000 Transfection Kit. Live cell imaging was performed as described above with the following modifications. Cells were illuminated in the GFP channel for Cry2 activation by blue light and imaged in the TxRed channel to monitor optodroplet formation. Before activation, we took images at the TxRed channel at 10 s intervals for 2 min. We then switched to the GFP channel and illuminated cells with blue light for 30 s, 60 s, and 90 s or 2 s, 5 s, and 10 s, as indicated in Fig. 6. After each noted duration of illumination, we acquired images of mCherry-fusion proteins in the TxRed channel.

FRAP of optodroplets was performed at an Olympus FV3000 confocal microscope with a 100× oil-immersion objective. Med1_(948-1157)_-mCherry-Cry2 and NLS*-Med15_(71-617)_-mCherry-Cry2 optodroplets were induced with blue light for 2 min, while Med15_(618-789)_-mCherry-Cry2 optodroplets were induced with blue light for 30 s. Optodroplets were photobleached with a 561 nm laser at 3% laser power for 1 s and post-bleach images were acquired at 3.22 s intervals for 30–40 cycles in the absence of 488 nm laser stimulation. Imaging settings were as follows: 12-bit image depth, 1024 × 1024 frame size.

### Gene expression analysis of IEGs during serum starvation

NIH3T3 cells under serum starvation were obtained by replacing the normal culture medium with DMEM medium containing 0.2% NCS and culturing for 24 h. Serum-starved cells were treated with 10% or 0.5% 1, 6-Hexanediol diluted in the starvation media for 1 min. Serum-starved cells with or without Hexanediol treatment were stimulated with DMEM medium containing 20% NCS for 15 min, 30 min, 60 min and 120 min. For Med15 knockdown experiment, U2OS cells were plated on 6-well plates at a density of 1 × 10^5^ cells/well and transfected with 200 nmol/L control siRNA or Med15 siRNA for 48 h. Transfected cells were incubated in growth medium containing 0.2% FBS and cultured for 24 h, and then stimulated with growth medium containing 20% FBS for 30 min or 60 min.

Total RNA was collected from about 5 × 10^6^ cells at each time point using the ReliaPrep RNA Miniprep System (Promega, Z6011). RNA was reverse transcribed using RevertAid First Strand cDNA Synthesis Kit (Thermo Fisher, K1622) following the manufacturer’s instructions. Quantitative real-time PCR was performed using SYBR Green master mix. Glyceraldehyde-3-phosphate dehydrogenase (*GAPDH*) mRNA was used as an internal control and IEG expression was measured before serum induction and at 15 min, 30 min, 60 min, and 120 min after serum induction. Primer sequences used for real-time PCR are described in Additional file 6: Table S4.

## Supplementary Information

**Additional file 1: **Fig. S1-S19. **Fig. S1** Med1 nuclear foci were disrupted by Hexanediol treatment and restored upon withdrawal. **Fig. S2** Characterization of Med15 nuclear foci. **Fig. S3** Med15 foci in mitotic cells. **Fig. S4** Response of Med1 and Med15 nuclear foci to Med15 depletion. **Fig. S5** Time lapse images of a T24 cell stably expressing GFP-hMed15 upon Hexanediol treatment and withdrawal. **Fig. S6** Dynamics of TagRFP-Med15 at nuclear foci in living cells. **Fig. S7** Time lapse images of GFP-hMed15 foci undergoing fusion and fission events. **Fig. S8** DYRK3 inhibition restores Med1 foci in some mitotic cells. **Fig. S9** Effects of DYRK3 overexpression on Med1 nuclear foci in NIH3T3 cells. **Fig. S10** Expression levels of TagRFP-DYRK3 affect the dissolution of GFP-Med15 foci. **Fig. S11** Displacement of overexpressed Med1 IDR from nucleolar regions upon expressing DYRK3. **Fig. S12** Representative images of NIH3T3 cells displaying nuclear foci formed by Med15 mutants. **Fig. S13** Formation of nuclear condensates by human Med15 truncation mutants in U2OS cells. **Fig. S14** Dynamics of optodroplets formed by Med1 and Med15 regions. **Fig. S15** Response of Med1 and Med15 nuclear foci to 10% Hexanediol treatment and withdrawal in the serum response experiment. **Fig. S16** Time lapse images of serum-starved T24 cells stably expressing GFP-hMed15 upon 0.5% Hexanediol treatment followed by 20% serum stimulation without Hexanediol. **Fig. S17** Effects of 10% Hexanediol treatment on IEG activation during serum response. **Fig. S18** Effects of Med15 knockdown on IEG activation during serum response in U2OS cells. **Fig. S19** Original western blot images.

**Additional file 2: Video S1.** Time lapse images of a cell from T24 stable cell line expressing GFP-hMed15 that was treated with 0.5% 1,6-Hexanediol and upon Hexanediol withdrawal. Images were taken every 10 s. Time points on the video are in mm:ss format. 0.5% Hexanediol was added at 3:40 and was replaced with fresh growth media at 12:40.

**Additional file 3: Video S2.** Time lapse images of a T24 cell transfected with GFP-Med15 in which Med15 foci were observed to undergo fusion events. Images were taken every 10 s. Time points on the video are in mm:ss format.

**Additional file 4: Video S3.** Time lapse images of a T24 cell transfected with GFP-Med15 in which Med15 foci were observed to undergo fission events. Images were taken every 10 s. Time points on the video are in mm:ss format.

**Additional file 5: Video S4.** Time lapse images of a cell from T24 stable cell line expressing GFP-hMed15 that was treated with 0.5% 1,6-Hexanediol followed by 20% serum stimulation. Images were taken every 10 s. Time points on the video are in mm:ss format. 0.5% Hexanediol was added at 4:00 and was replaced with growth media containing 20% serum at 16:10.

**Additional file 6: Table S1-S4**.**Table S1** Primer sequences used to clone cDNA and mouse Med15 truncation mutants. **Table S2** Primer sequences used to clone human Med15 and truncation mutants. **Table S3** Primer sequences used to clone the lentiviral vectors. **Table S4** Primer sequences used for real-time PCR.

## Data Availability

All data analyzed in this study are included in this article and additional files.

## Abbreviations

FRAP: Fluorescence recovery after photobleaching
IDR: Intrinsically disordered region
IEG: Immediate early genes
LLPS: Liquid-liquid phase separation
NLS: Nuclear localization signal
Pol II: RNA polymerase II
TF: Transcription factor

## References

[1] Hyman AA, Weber CA, Julicher F (2014). Liquid-liquid phase separation in biology. Annu Rev Cell Dev Biol.

[2] Gomes E, Shorter J (2019). The molecular language of membraneless organelles. J Biol Chem.

[3] Alberti S, Gladfelter A, Mittag T (2019). Considerations and challenges in studying liquid-liquid phase separation and biomolecular condensates. Cell.

[4] Banani SF, Lee HO, Hyman AA, Rosen MK (2017). Biomolecular condensates: organizers of cellular biochemistry. Nat Rev Mol Cell Biol.

[5] Shin Y, Brangwynne CP. Liquid phase condensation in cell physiology and disease. Science. 2017;357(6357):eaaf4382. 10.1126/science.aaf4382 10.1126/science.aaf4382 28935776

[6] Chong S, Dugast-Darzacq C, Liu Z, Dong P, Dailey GM, Cattoglio C, et al. Imaging dynamic and selective low-complexity domain interactions that control gene transcription. Science. 2018;361(6400):eaar2555. 10.1126/science.aar2555 10.1126/science.aar2555 PMC6961784 29930090

[7] Kato M, Han TW, Xie S, Shi K, Du X, Wu LC, Mirzaei H, Goldsmith EJ, Longgood J, Pei J (2012). Cell-free formation of RNA granules: low complexity sequence domains form dynamic fibers within hydrogels. Cell.

[8] Lin Y, Currie SL, Rosen MK (2017). Intrinsically disordered sequences enable modulation of protein phase separation through distributed tyrosine motifs. J Biol Chem.

[9] Rai AK, Chen JX, Selbach M, Pelkmans L (2018). Kinase-controlled phase transition of membraneless organelles in mitosis. Nature.

[10] Hnisz D, Shrinivas K, Young RA, Chakraborty AK, Sharp PA (2017). A phase separation model for transcriptional control. Cell.

[11] Lu H, Yu D, Hansen AS, Ganguly S, Liu R, Heckert A, Darzacq X, Zhou Q (2018). Phase-separation mechanism for C-terminal hyperphosphorylation of RNA polymerase II. Nature.

[12] Guo YE, Manteiga JC, Henninger JE, Sabari BR, Dall'Agnese A, Hannett NM, Spille JH, Afeyan LK, Zamudio AV, Shrinivas K (2019). Pol II phosphorylation regulates a switch between transcriptional and splicing condensates. Nature.

[13] Sabari BR, Dall'Agnese A, Boija A, Klein IA, Coffey EL, Shrinivas K, et al. Coactivator condensation at super-enhancers links phase separation and gene control. Science. 2018;361(6400):eaar3958. 10.1126/science.aar3958. 10.1126/science.aar3958 PMC6092193 29930091

[14] Cho WK, Spille JH, Hecht M, Lee C, Li C, Grube V, Cisse II (2018). Mediator and RNA polymerase II clusters associate in transcription-dependent condensates. Science.

[15] Boija A, Klein IA, Sabari BR, Dall'Agnese A, Coffey EL, Zamudio AV, Li CH, Shrinivas K, Manteiga JC, Hannett NM (2018). Transcription factors activate genes through the phase-separation capacity of their activation domains. Cell.

[16] Soutourina J (2018). Transcription regulation by the Mediator complex. Nat Rev Mol Cell Biol.

[17] Harper TM, Taatjes DJ (2018). The complex structure and function of Mediator. J Biol Chem.

[18] Yang F, Vought BW, Satterlee JS, Walker AK, Jim Sun ZY, Watts JL, DeBeaumont R, Saito RM, Hyberts SG, Yang S (2006). An ARC/Mediator subunit required for SREBP control of cholesterol and lipid homeostasis. Nature.

[19] Cooper DG, Fassler JS (2019). Med15: glutamine-rich mediator subunit with potential for plasticity. Trends Biochem Sci.

[20] Tuttle LM, Pacheco D, Warfield L, Luo J, Ranish J, Hahn S, Klevit RE (2018). Gcn4-mediator specificity is mediated by a large and dynamic fuzzy protein-protein complex. Cell Rep.

[21] Zhu X, Chen L, Carlsten JO, Liu Q, Yang J, Liu B, Gustafsson CM (2015). Mediator tail subunits can form amyloid-like aggregates in vivo and affect stress response in yeast. Nucleic Acids Res.

[22] Lionnet T, Czaplinski K, Darzacq X, Shav-Tal Y, Wells AL, Chao JA, Park HY, de Turris V, Lopez-Jones M, Singer RH (2011). A transgenic mouse for in vivo detection of endogenous labeled mRNA. Nat Methods.

[23] Spector DL, Fu XD, Maniatis T (1991). Associations between distinct pre-mRNA splicing components and the cell nucleus. EMBO J.

[24] Nizami Z, Deryusheva S, Gall JG (2010). The Cajal body and histone locus body. Cold Spring Harb Perspect Biol.

[25] Hernandez-Verdun D (2011). Assembly and disassembly of the nucleolus during the cell cycle. Nucleus.

[26] Nagulapalli M, Maji S, Dwivedi N, Dahiya P, Thakur JK (2016). Evolution of disorder in Mediator complex and its functional relevance. Nucleic Acids Res.

[27] Duster R, Kaltheuner IH, Schmitz M, Geyer M (2021). 1,6-Hexanediol, commonly used to dissolve liquid-liquid phase separated condensates, directly impairs kinase and phosphatase activities. J Biol Chem.

[28] Maharana S, Wang J, Papadopoulos DK, Richter D, Pozniakovsky A, Poser I, Bickle M, Rizk S, Guillen-Boixet J, Franzmann TM (2018). RNA buffers the phase separation behavior of prion-like RNA binding proteins. Science.

[29] Shin Y, Berry J, Pannucci N, Haataja MP, Toettcher JE, Brangwynne CP (2017). Spatiotemporal control of intracellular phase transitions using light-activated optoDroplets. Cell.

[30] Bahrami S, Drablos F (2016). Gene regulation in the immediate-early response process. Adv Biol Regul.

[31] Healy S, Khan P, Davie JR (2013). Immediate early response genes and cell transformation. Pharmacol Ther.

[32] Li P, Banjade S, Cheng HC, Kim S, Chen B, Guo L, Llaguno M, Hollingsworth JV, King DS, Banani SF, Russo PS, Jiang QX, Nixon BT, Rosen MK (2012). Phase transitions in the assembly of multivalent signalling proteins. Nature.

[33] McSwiggen DT, Hansen AS, Teves SS, Marie-Nelly H, Hao Y, Heckert AB, Umemoto KK, Dugast-Darzacq C, Tjian R, Darzacq X (2019). Evidence for DNA-mediated nuclear compartmentalization distinct from phase separation. Elife.

[34] Narlikar GJ, Myong S, Larson D, Maeshima K, Francis N, Rippe K, Sabari B, Strader L, Tjian R (2021). Is transcriptional regulation just going through a phase?. Mol Cell.

[35] Sawyer IA, Bartek J, Dundr M (2019). Phase separated microenvironments inside the cell nucleus are linked to disease and regulate epigenetic state, transcription and RNA processing. Semin Cell Dev Biol.

[36] Zhong L, Liao D, Zhang M, Zeng C, Li X, Zhang R, Ma H, Kang T (2019). YTHDF2 suppresses cell proliferation and growth via destabilizing the EGFR mRNA in hepatocellular carcinoma. Cancer Lett.

