# Supplementary material for: Formation of nuclear condensates by the Mediator complex subunit Med15 in mammalian cells
Source: BMC Biol. 2021 Nov 17;19:245. doi: 10.1186/s12915-021-01178-y (PMC8597291; doi:10.1186/s12915-021-01178-y)
Supplement: Supplementary file 1 — Additional file 1: Fig. S1-S19. Fig. S1 Med1 nuclear foci were disrupted by Hexanediol treatment and restored upon withdrawal. Fig. S2 Characterization of Med15 nuclear foci. Fig. S3 Med15 foci in mitotic cells. Fig. S4 Response of Med1 and Med15 nuclear foci to Med15 depletion. Fig. S5 Time lapse images of a T24 cell stably expressing GFP-hMed15 upon Hexanediol treatment and withdrawal. Fig. S6 Dynamics of TagRFP-Med15 at nuclear foci in living cells. Fig. S7 Time lapse images of GFP-hMed15 foci undergoing fusion and fission events. Fig. S8 DYRK3 inhibition restores Med1 foci in some mitotic cells. Fig. S9 Effects of DYRK3 overexpression on Med1 nuclear foci in NIH3T3 cells. Fig. S10 Expression levels of TagRFP-DYRK3 affect the dissolution of GFP-Med15 foci. Fig. S11 Displacement of overexpressed Med1 IDR from nucleolar regions upon expressing DYRK3. Fig. S12 Representative images of NIH3T3 cells displaying nuclear foci formed by Med15 mutants. Fig. S13 Formation of nuclear condensates by human Med15 truncation mutants in U2OS cells. Fig. S14 Dynamics of optodroplets formed by Med1 and Med15 regions. Fig. S15 Response of Med1 and Med15 nuclear foci to 10% Hexanediol treatment and withdrawal in the serum response experiment. Fig. S16 Time lapse images of serum-starved T24 cells stably expressing GFP-hMed15 upon 0.5% Hexanediol treatment followed by 20% serum stimulation without Hexanediol. Fig. S17 Effects of 10% Hexanediol treatment on IEG activation during serum response. Fig. S18 Effects of Med15 knockdown on IEG activation during serum response in U2OS cells. Fig. S19 Original western blot images. [file 12915_2021_1178_MOESM1_ESM.pdf]

Formation of Nuclear Condensates by the Mediator Complex Subunit Med15 in Mammalian Cells

Yuanyuan Shi[1], Jian Chen[1], Wei-jie Zeng[1], Miao Li[1], Wenxue Zhao[1], Xing-Ding Zhang[1*], Jie Yao[1,2*]

[1]Molecular Cancer Research Center, School of Medicine, Shenzhen Campus of Sun Yat-sen University, Sun Yat-sen University, Shenzhen, China
[2]Present Address: Allen Institute for Cell Science, Seattle, WA, 98109, USA
[*]Corresponding authors, Email: zhangxd39@mail.sysu.edu.cn, jie.yao@alleninstitute.org

Additional file 1. Supplementary figures S1-S19

Fig. S1 Med1 nuclear foci were disrupted by Hexanediol treatment and restored upon withdrawal.

Fig. S2 Characterization of Med15 nuclear foci.

Fig. S3 Med15 foci in mitotic cells.

Fig. S4 Response of Med1 and Med15 nuclear foci to Med15 depletion.

Fig. S5 Time lapse images of a T24 cell stably expressing GFP-hMed15 upon Hexanediol treatment and withdrawal.

Fig. S6 Dynamics of TagRFP-Med15 at nuclear foci in living cells.

Fig. S7 Time lapse images of GFP-hMed15 foci undergoing fusion and fission events.

Fig. S8 DYRK3 inhibition restores Med1 foci in some mitotic cells.

Fig. S9 Effects of DYRK3 overexpression on Med1 nuclear foci in NIH3T3 cells.

Fig. S10 Expression levels of TagRFP-DYRK3 affect the dissolution of GFP-Med15 foci.

Fig. S11 Displacement of overexpressed Med1 IDR from nucleolar regions upon expressing DYRK3.

Fig. S12 Representative images of NIH3T3 cells displaying nuclear foci formed by Med15 mutants.

Fig. S13 Formation of nuclear condensates by human Med15 truncation mutants in U2OS cells.

Fig. S14 Dynamics of optodroplets formed by Med1 and Med15 regions.

Fig. S15 Response of Med1 and Med15 nuclear foci to 10% Hexanediol treatment and withdrawal in the serum response experiment.

Fig. S16 Time lapse images of serum-starved T24 cells stably expressing GFP-hMed15 upon 0.5% Hexanediol treatment followed by 20% serum stimulation without Hexanediol.

Fig. S17 Effects of 10% Hexanediol treatment on IEG activation during serum response.

Fig. S18 Effects of Med15 knockdown on IEG activation during serum response in U2OS cells.

Fig. S19 Original western blot images.

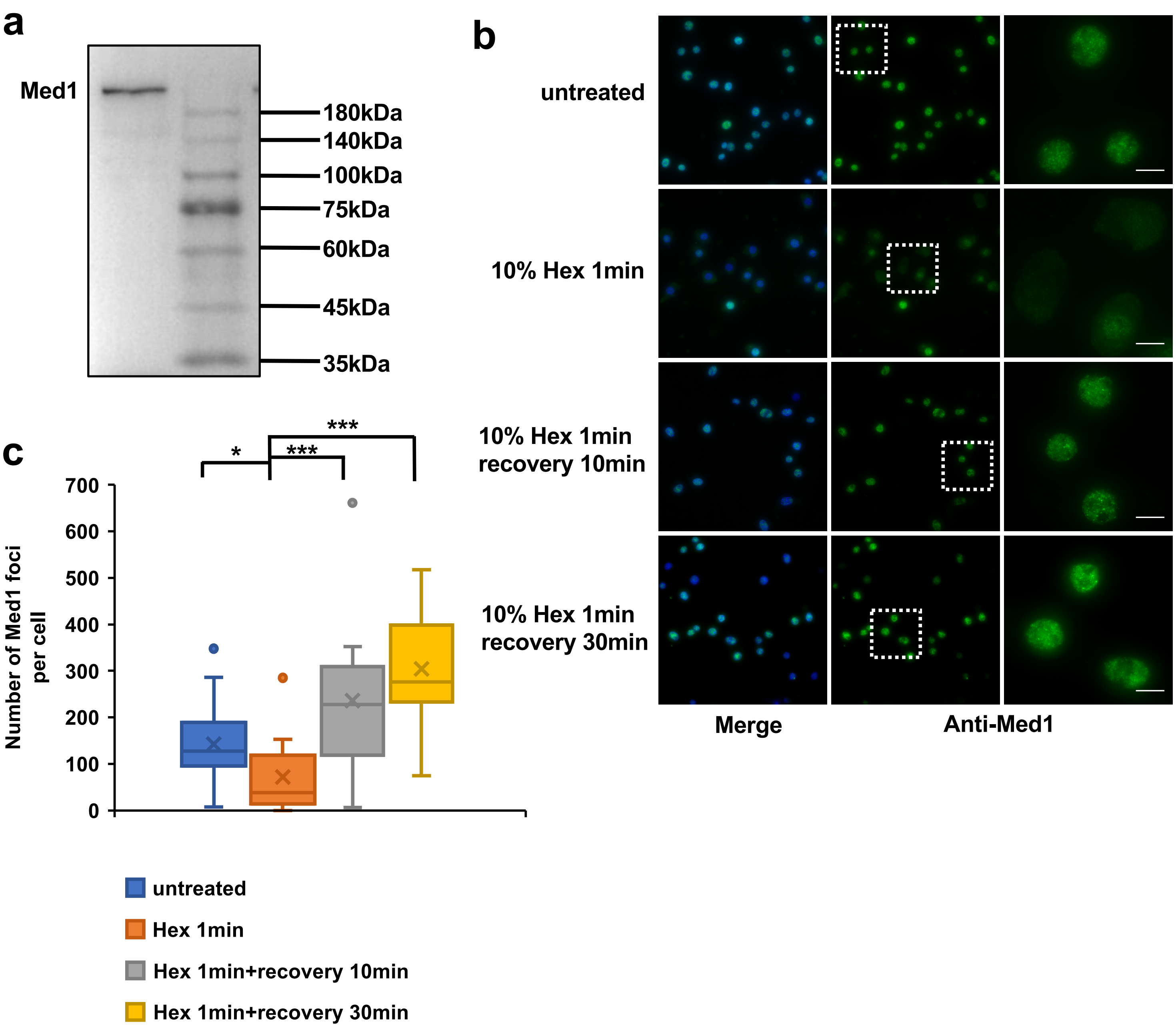

Fig. S1 Med1 nuclear foci were disrupted by Hexanediol treatment and restored upon withdrawal. a Detecting endogenous human Med1 protein in U2OS cell extracts by western blot. **b** Fluorescence images of NIH3T3 cells stained with Hoechst33342 (blue) and an anti-Med1 antibody (green) without treatment (first row), after 1 min treatment with 10% 1,6-Hexanediol (second row), and after 1 min 10% Hexanediol treatment followed by recovery in normal culture media for 10 min (third row) or 30 min (fourth row). Left column: merged images; Middle column: anti-Med1; Right column: enlarged images of the square areas marked with white borders (middle column). Scale bars: 10 μm. Similar results were obtained from three independent experiments. **c** The number of Med1 foci from individual cells at treatment conditions described in b quantified by AirLocalize (Intensity threshold: 1000). The numbers of analyzed cells were 19, 17, 18 and 18, respectively. * and *** indicate $p < 0.05$ and $p < 0.001$ in student's t-test, respectively.

Fig. S2

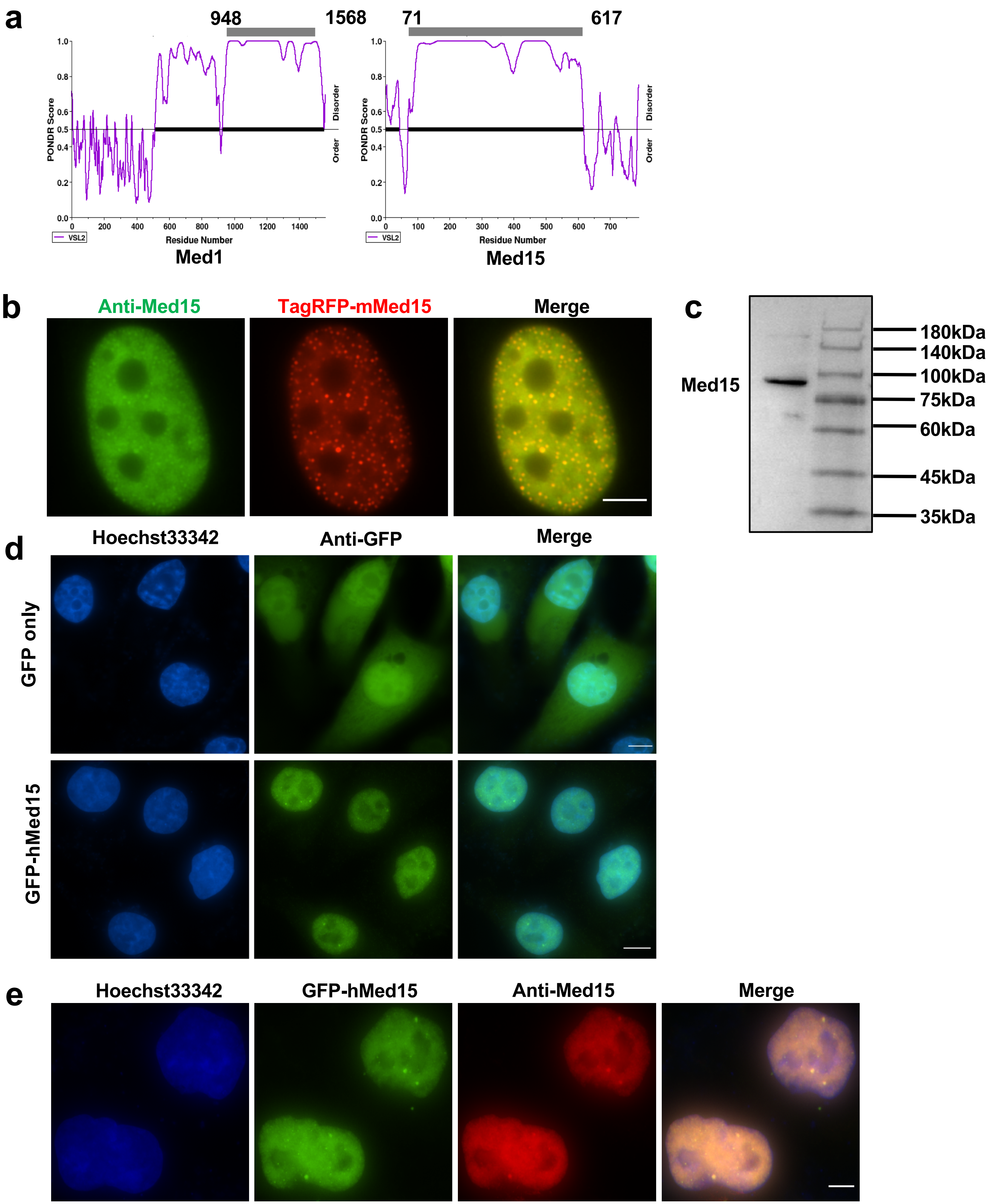
a
948
1568
71
617
PONDR Score
Disorder
Order
Residue Number
VSL2
Med1
Med15
b
Anti-Med15
TagRFP-mMed15
Merge
c
Med15
180kDa
140kDa
100kDa
75kDa
60kDa
45kDa
35kDa
d
Hoechst33342
Anti-GFP
Merge
GFP only
GFP-hMed15
e
Hoechst33342
GFP-hMed15
Anti-Med15
Merge

Fig. S2 Characterization of Med15 nuclear foci. a Graphs plotting intrinsic disorder regions of mouse Med1 and Med15. PONDR (Predictor of Natural Disordered Regions) VSL2 scores are shown on the y axes, and amino acid positions are shown on the x axes. Grey bars indicate the IDRs studied in this work. **b** Wide-field fluorescence images of U2OS cells transfected with TagRFP-mMed15 (red) and stained with anti-Med15 (green). Similar results were obtained from three independent experiments. **c** Detecting endogenous human Med15 protein in U2OS cell extracts by western blot. **d** Wide-field fluorescence images of human T24 cell lines stably expressing GFP only (upper row) or GFP-human Med15 (lower row). Cells were co-stained with an anti-GFP antibody (green) and Hoechst33342 (blue). **e** Wide-field fluorescence images of a stable human T24 cell line expressing GFP-Med15(green) co-stained with an anti-Med15 antibody (red) and Hoechst33342 (blue). Scale bars are 10 μm in d and 5 μm in b and e.

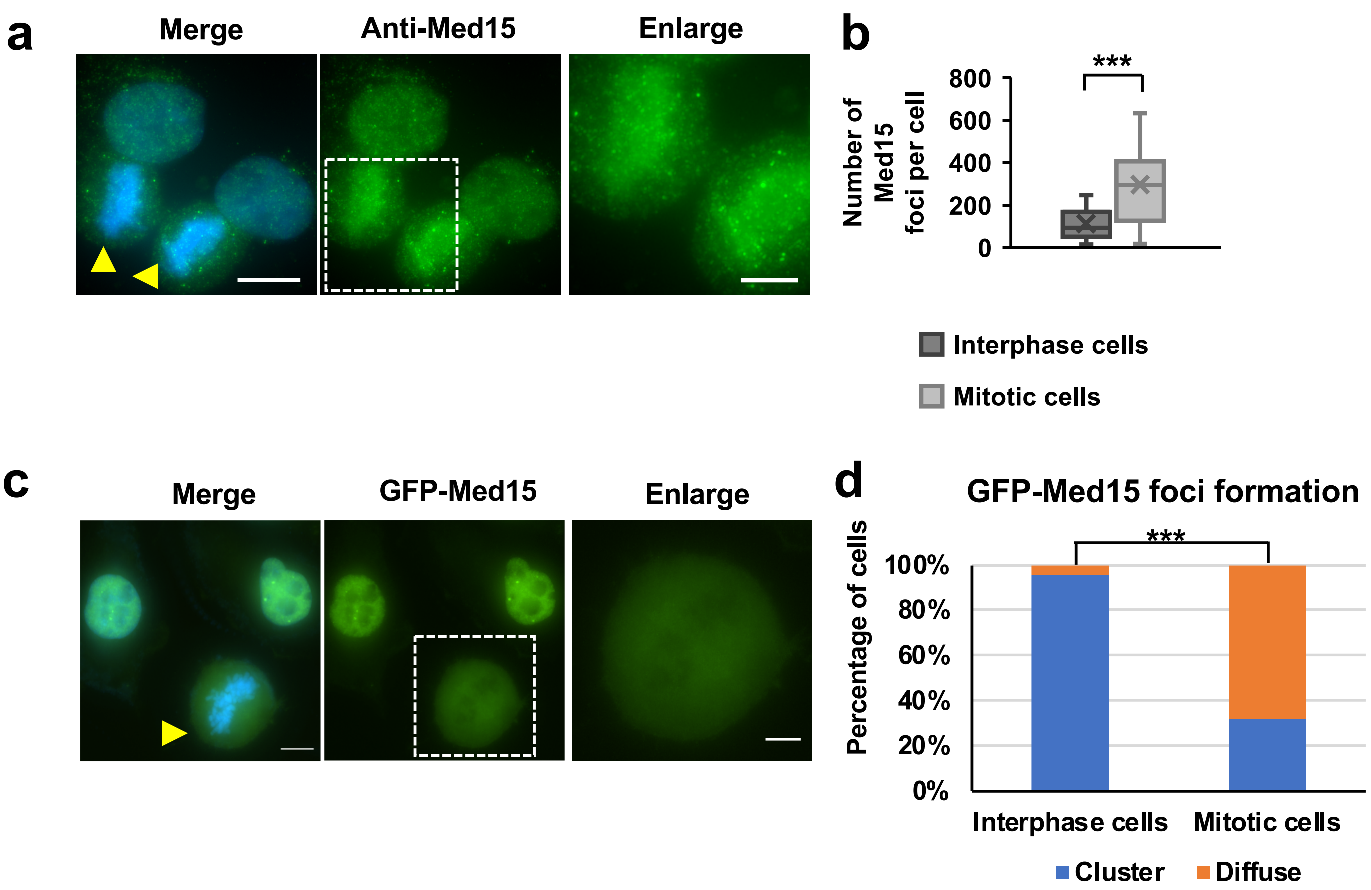

Fig. S3 Med15 foci in mitotic cells. a Interphase and mitotic U2OS cells co-stained with Hoechst33342 (blue) and an anti-Med15 antibody (green). **b** The number of Med15 foci quantified in interphase (n = 21) and mitotic U2OS cells (n =15) at a threshold of 500. **c** Interphase and mitotic cells of the T24 stable cell line expressing GFP-Med15. In a and c, yellow arrowheads indicate mitotic cells, and the right column shows the enlarged images of the areas marked with white border in the middle column. **d** Percentages of cells from the T24 stable cell line displaying diffuse localization vs clusters of GFP-Med15 in interphase (n = 67) vs mitotic cells (n = 40). In b and d, *** indicates $p < 0.001$. Scale bars are 10 μm in merged images and 5 μm in enlarged images.

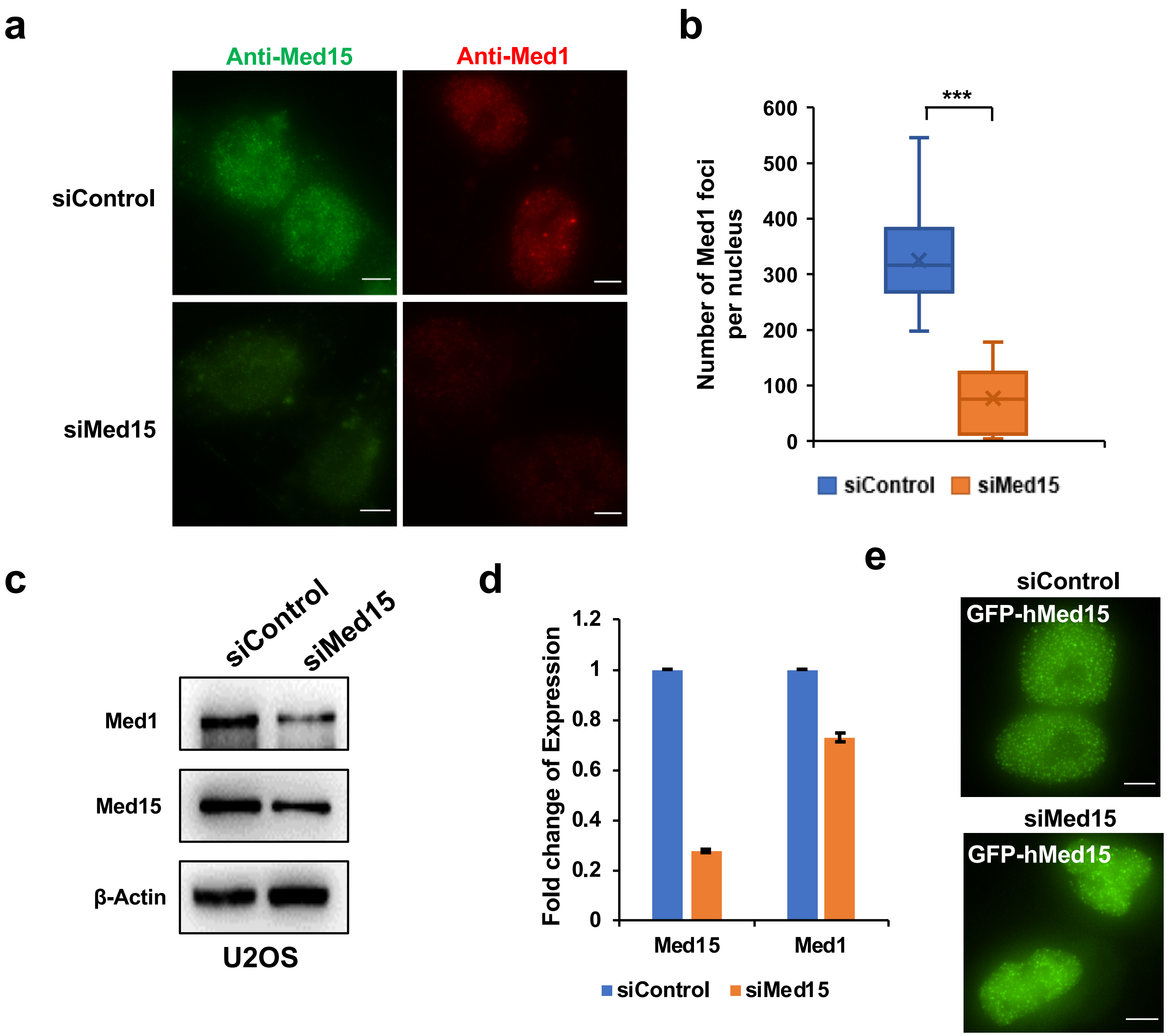

Fig. S4 Response of Med1 and Med15 nuclear foci to Med15 depletion. a Representative images of U2OS cells transfected with control siRNA and Med15 siRNA and stained with an anti-Med15 antibody (green) or an anti-Med1 antibody (red). Similar results were obtained from two independent experiments. **b** The number of Med1 foci in siControl and siMed15 cells. The numbers of analyzed cells were 20 and 17, respectively. Student's t-test: $p < 0.001$ (indicated by ***). **c** Detecting endogenous human Med15 and Med1 proteins in U2OS cells treated with control siRNA or Med15 siRNA by western blot. Similar results were obtained from three independent experiments. **d** Detecting Med1 and Med15 mRNA expression in siControl and siMed15 cells. Data are presented as the mean ± SEM, n = 3. **e** Representative images of GFP-hMed15 in U2OS cells transfected with control siRNA and Med15 siRNA. All scale bars: 5 μm.

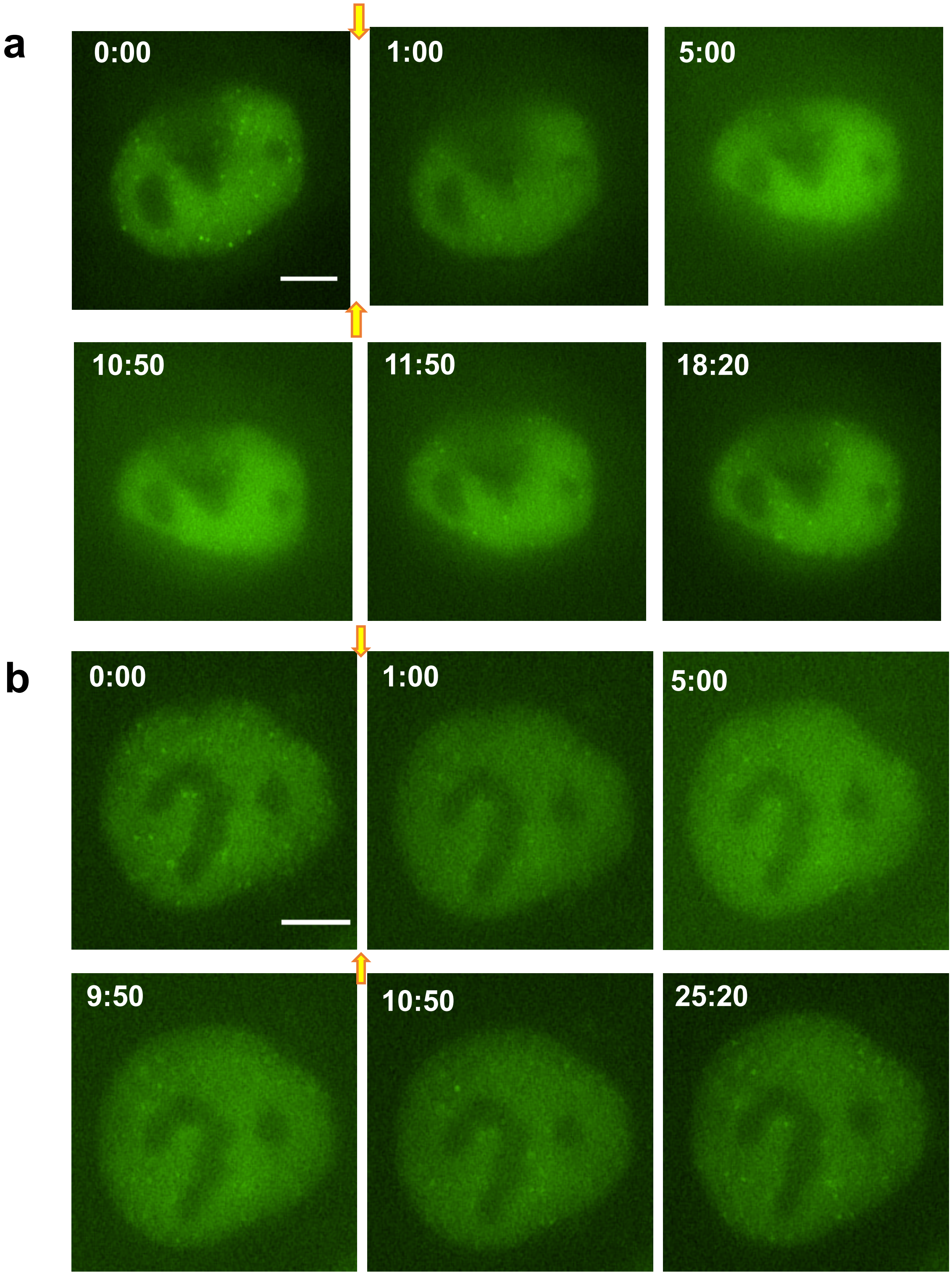

Fig. S5 Time lapse images of a T24 cell stably expressing GFP-hMed15 upon Hexanediol treatment and withdrawal. Yellow arrows pointing downwards and upwards indicate the timepoints of adding 0.5% Hexanediol and replacing with fresh growth media, respectively. Time points are in mm:ss format. Panels a and b indicate two different cells. Scale bars: 5 μm.

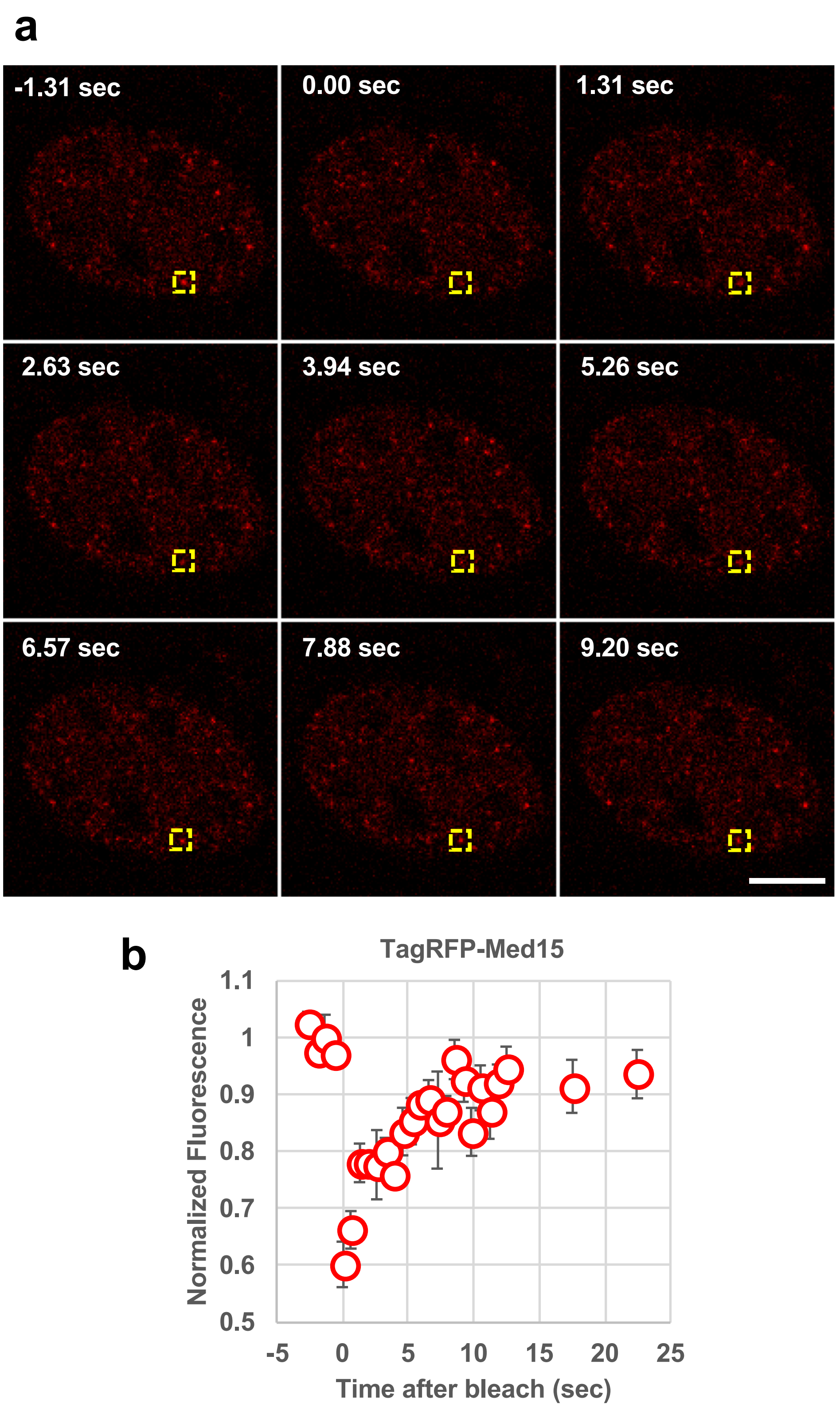

Fig. S6 Dynamics of TagRFP-Med15 at nuclear foci in living cells. a Time-lapse images of an NIH3T3 cell nucleus expressing TagRFP-mMed15 during a FRAP experiment. Yellow boxes indicate the photobleached area. t = 0.00 sec indicates the time point immediately after photobleaching. **b** Plot of TagRFP-mMed15 fluorescence intensity at the photobleached area within 25 seconds after photobleaching. The time intervals between individual frames in the first 20 cycles and the last 20 cycles of post-bleach were 655 ms and 5 s, respectively. Normalized fluorescence intensities are presented as mean ± SEM, n=4.

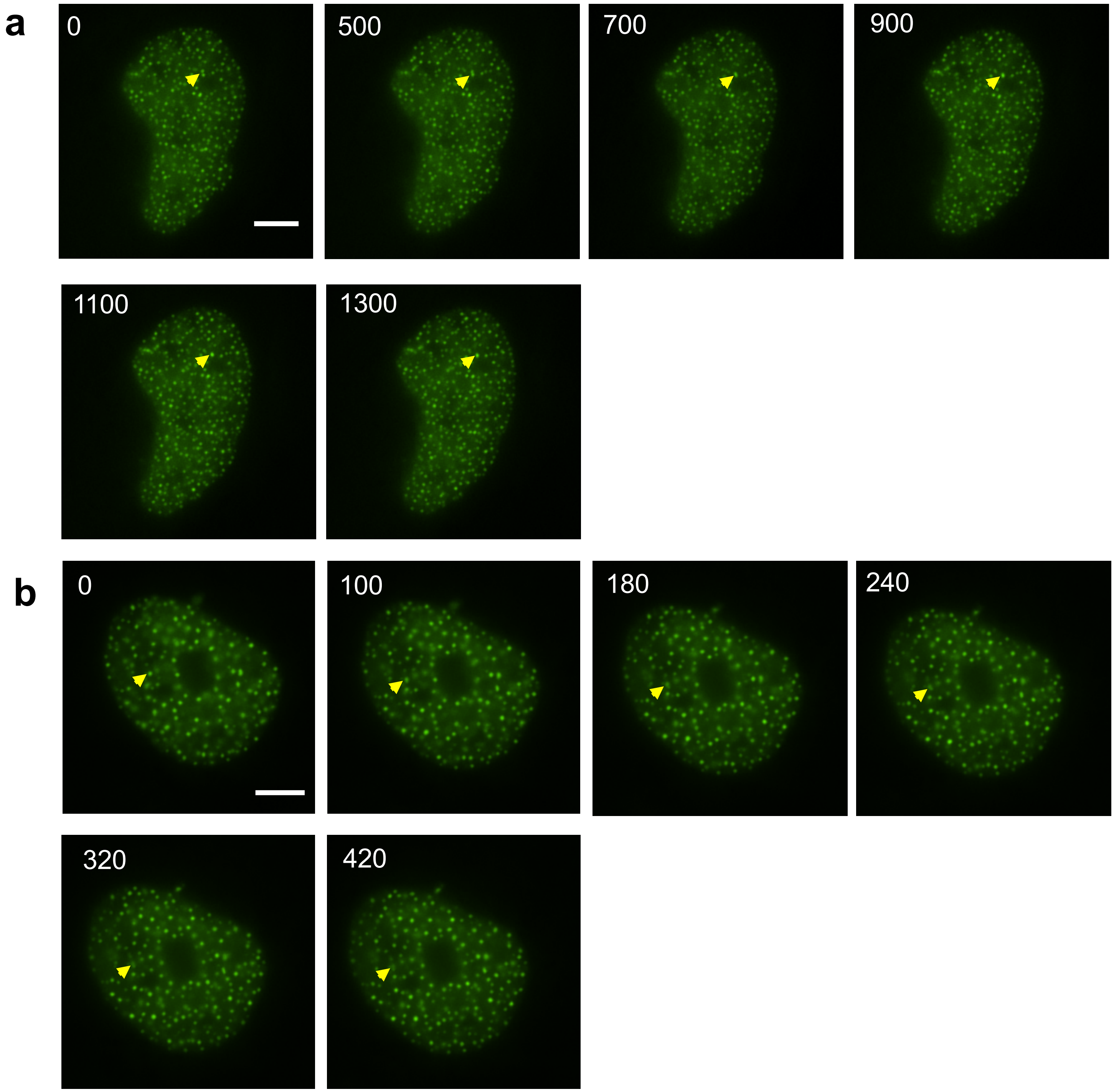

Fig. S7 Time lapse images of GFP-hMed15 foci undergoing fusion and fission events. Yellow arrowheads in panel a and b indicate the Med15 foci undergoing fusion or fission events, respectively. Scale bars: 5 μm. Noted time points are in seconds.

Fig. S8

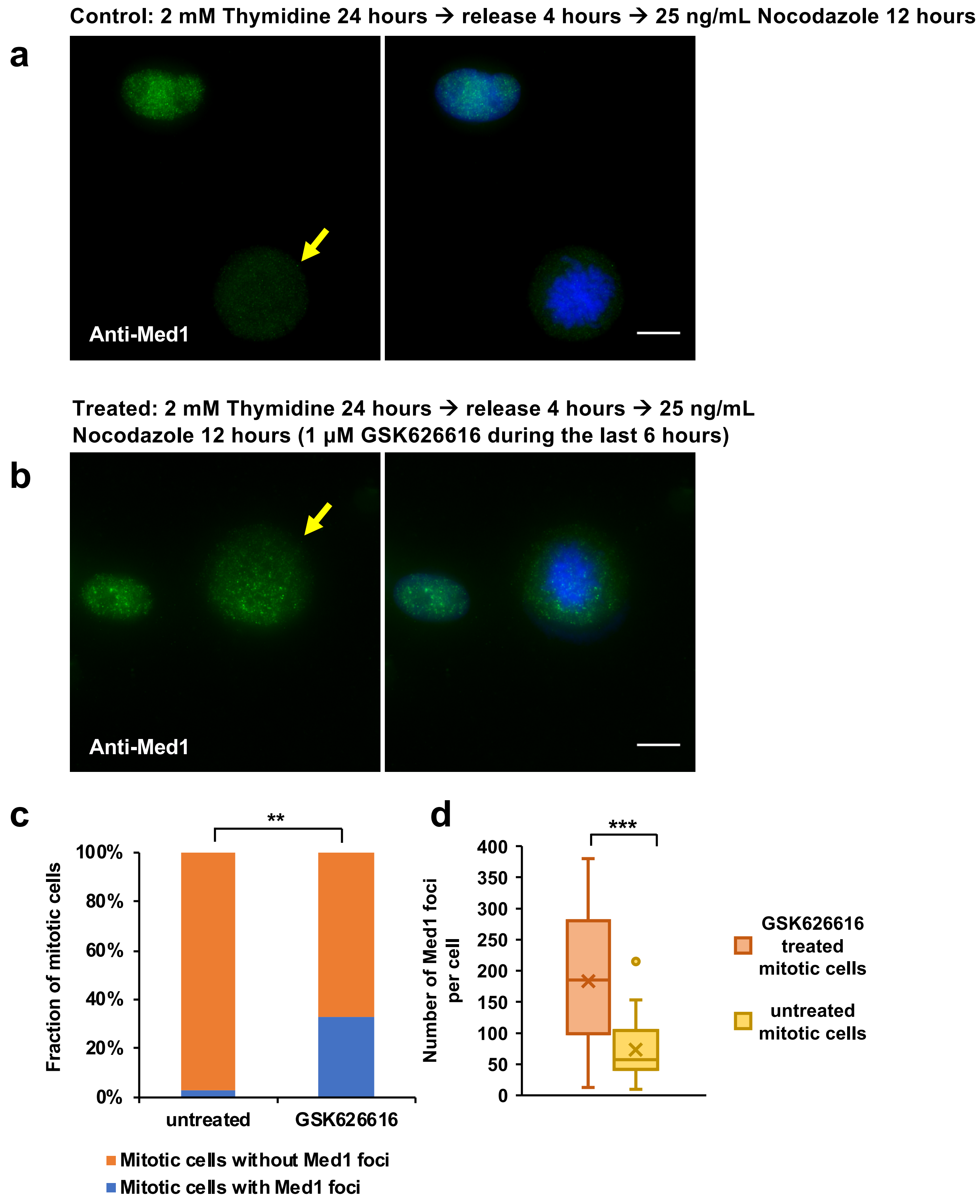

Control: 2 mM Thymidine 24 hours → release 4 hours → 25 ng/mL Nocodazole 12 hours
a
Anti-Med1
Treated: 2 mM Thymidine 24 hours → release 4 hours → 25 ng/mL
Nocodazole 12 hours (1 μM GSK626616 during the last 6 hours)
b
Anti-Med1
c
**
Fraction of mitotic cells
100%
80%
60%
40%
20%
0%
untreated
GSK626616
Mitotic cells without Med1 foci
Mitotic cells with Med1 foci
d
***
Number of Med1 foci per cell
400
350
300
250
200
150
100
50
0
GSK626616 treated mitotic cells
untreated mitotic cells

Fig. S8 DYRK3 inhibition restores Med1 foci in some mitotic cells. a, b Fluorescence images of U2OS cells stained with an anti-Med1 antibody (green) and Hoechst 33342 (blue). (a) Cells were treated with 2 mM Thymidine for 24 h, released for 4 h and treated with 25 ng/mL Nocodazole for 12 h. (b) The treatment was the same as in control cells except that 1 μM GSK626616 was added during the last 6 h of nocodazole treatment. Yellow arrows indicate mitotic cells. Scale bars: 10 μm. **c** Percentages of mitotic U2OS cells displaying Med1 foci in the control group (n = 33) and in the treatment group (n = 40). Fisher's exact test: $p < 0.01$ (indicated by **). **d** The number of Med1 clusters quantified in individual mitotic U2OS cells between the control group (n = 16) and treatment group (n = 17) (Intensity threshold: 400). Student's t-test: $p < 0.001$ (indicated by ***).

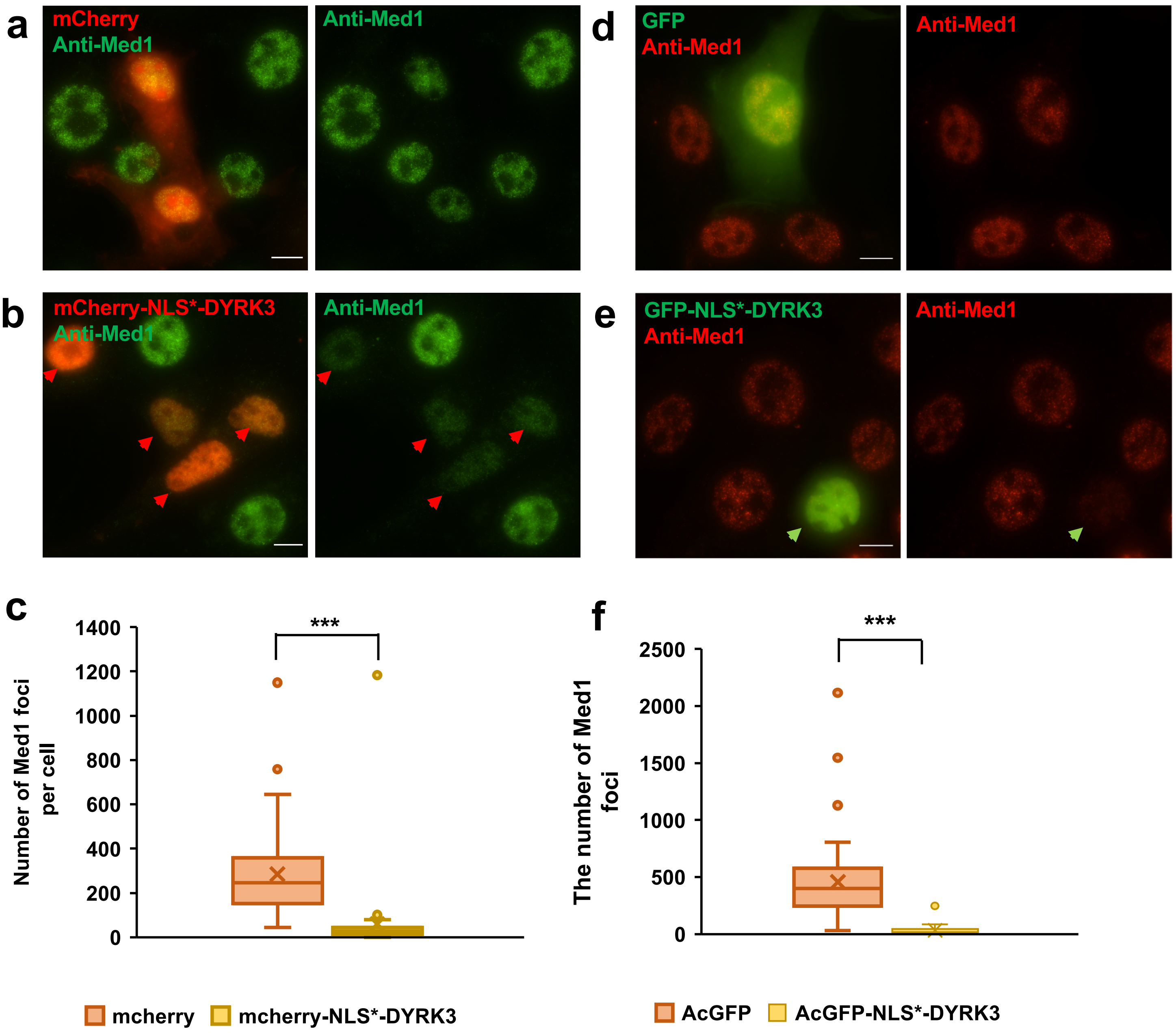

Fig. S9 Effects of DYRK3 overexpression on Med1 nuclear foci in NIH3T3 cells. a, b Fluorescence images of NIH3T3 cells transfected with mCherry (a, red) or mCherry-NLS*-DYRK3 (b, red) and stained with anti-Med1 antibody (green). Red arrowheads in b indicate transfected cells. **c** The number of Med1 clusters quantified in individual NIH3T3 cells transfected with mCherry (n = 67) or with mCherry-DYRK3 (n = 39) (Intensity threshold: 600). Student's t-test: $p < 0.001$ (indicated by ***). **d, e** Fluorescence images of NIH3T3 cells transfected with AcGFP (d, green) or AcGFP-NLS*-DYRK3 (e, green) and stained with anti-Med1 antibody (red). Green arrowheads in e indicate a transfected cell. **f** The number of Med1 clusters quantified in individual NIH3T3 cells transfected with AcGFP (n = 55) or with AcGFP-NLS*-DYRK3 (n = 54) (Intensity threshold: 600). Student's t-test: $p < 0.001$ (indicated by ***). All scale bars: 10 μm.

Fig. S10

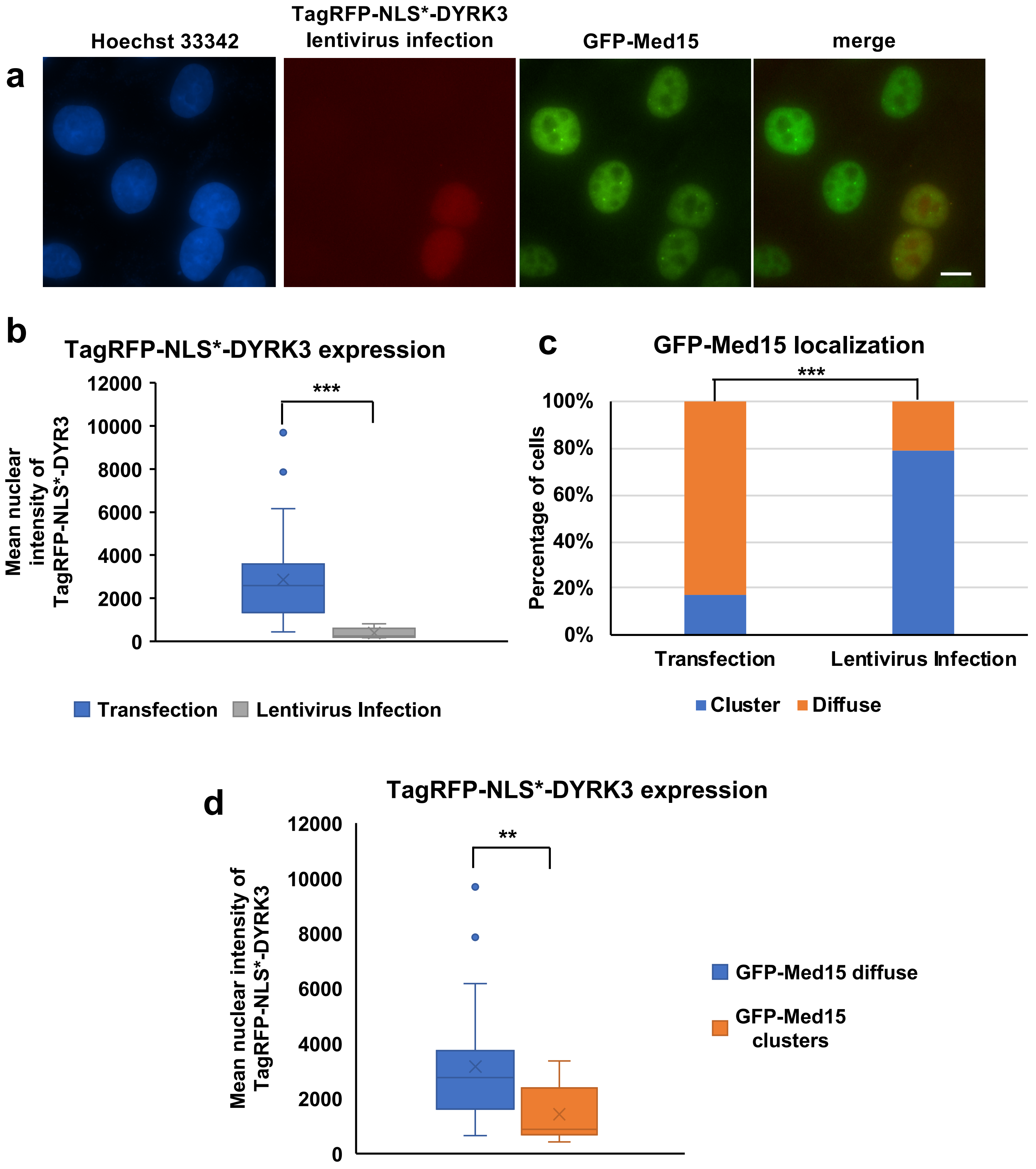

a
Hoechst 33342
TagRFP-NLS*-DYRK3
lentivirus infection
GFP-Med15
merge
b
TagRFP-NLS*-DYRK3 expression
Mean nuclear
intensity of
TagRFP-NLS*-DYR3
12000
10000
8000
6000
4000
2000
0
***
Transfection
Lentivirus Infection
c
GFP-Med15 localization
***
Percentage of cells
100%
80%
60%
40%
20%
0%
Transfection
Lentivirus Infection
Cluster
Diffuse
d
TagRFP-NLS*-DYRK3 expression
Mean nuclear intensity of
TagRFP-NLS*-DYRK3
12000
10000
8000
6000
4000
2000
0
**
GFP-Med15 diffuse
GFP-Med15
clusters

Fig. S10 Expression levels of TagRFP-DYRK3 affect the dissolution of GFP-Med15 foci. a Fluorescence images of the T24 stable cell line infected with lentivirus harboring pSin-EF2-TagRFP-NLS*-DYRK3 expression vector. **b** Box plot of mean fluorescence intensity of TagRFP-NLS*-DYRK3 in the mid-section of the nucleus in transfected T24 cells (n = 57) and in lentivirus infected T24 cells (n = 14). The same excitation intensity was applied in imaging all cells. Exposure time was 900ms in the RFP channel and digital gain was set to 11.4 for all cells. *** indicates $p < 0.001$ in student's t-test (unpaired). **c** Percentages of cells displaying diffuse localization vs clusters of GFP-Med15 in transfected T24 cells (n = 57) and in lentivirus infected T24 cells (n = 14). *** indicates $p < 0.001$ in Fisher's exact test. **d** Box plot of mean fluorescence intensity of TagRFP-NLS*-DYRK3 in the mid-section of the nucleus in transfected T24 cells displaying diffuse localization (n = 47) vs clusters (n = 10) of GFP-Med15. ** indicates $p < 0.01$ in student's t-test (unpaired). Excitation intensity, exposure time and digital gain were identical for all cells.

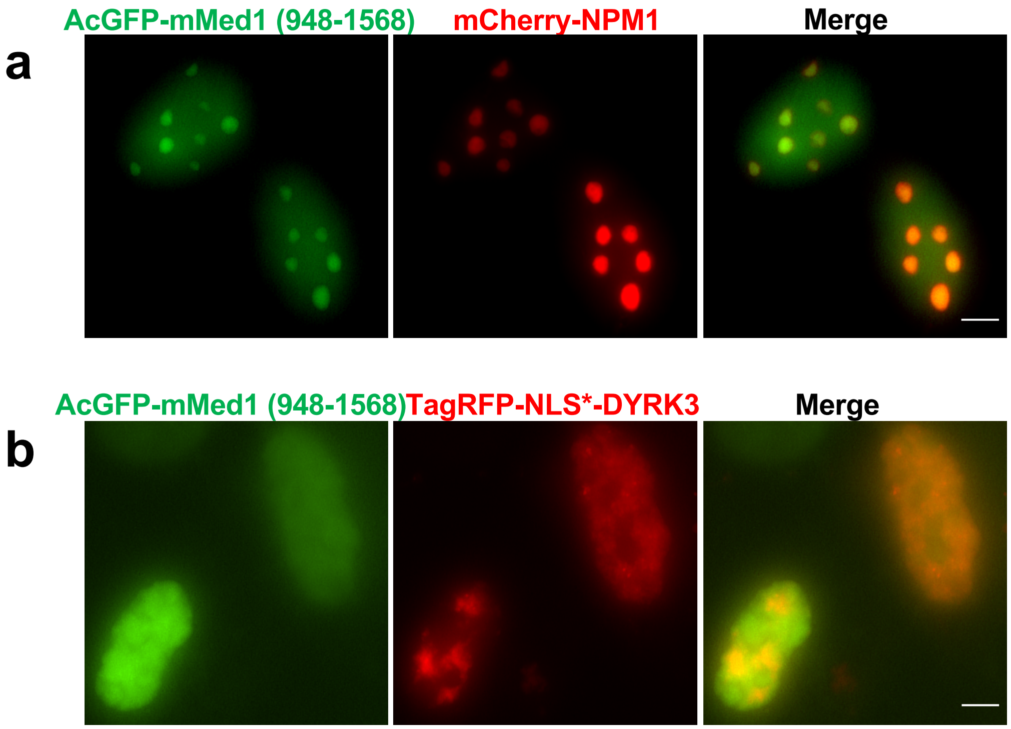

Fig. S11 Displacement of overexpressed Med1 IDR from nucleolar regions upon expressing DYRK3. a Fluorescence images of NIH3T3 cells (n=15) co-transfected with AcGFP-mMed1 (948-1568) (green) and mCherry-NPM1 (red). **b** Fluorescence images of NIH3T3 cells (n=15) co-transfected with AcGFP-mMed1(948-1568) (green) and TagRFP-NLS*-DYRK3 (red). Scale bars are 5 μm.

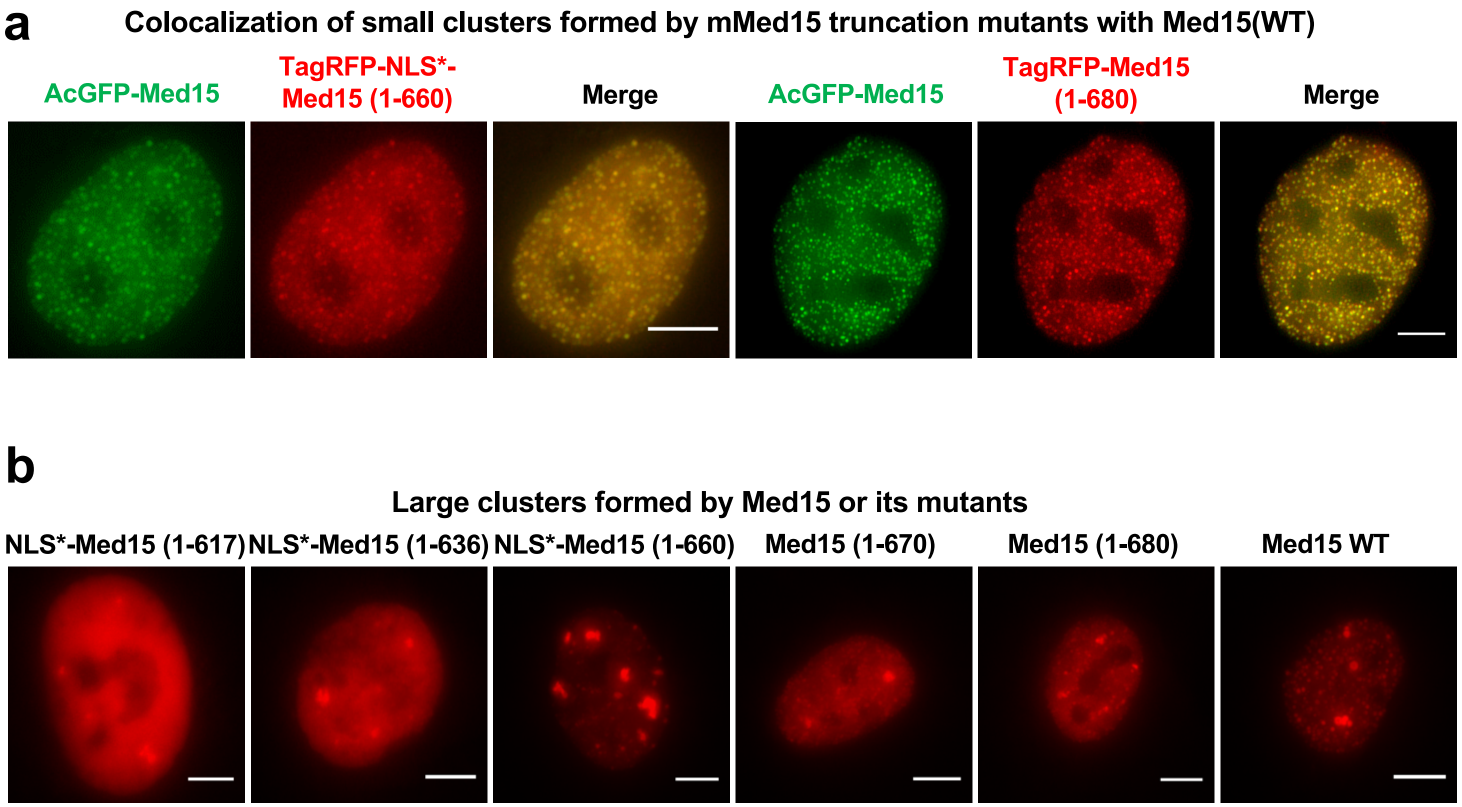

Fig. S12 Representative images of NIH3T3 cells displaying nuclear foci formed by Med15 mutants. a Fluorescence images of an NIH3T3 cell transfected with AcGFP-Med15 and TagRFP-NLS*-Med15 (1-660) (left) or with AcGFP-Med15 and TagRFP-Med15 (1-680) (Right). **b** Representative images of NIH3T3 cells displaying large nuclear foci of Med15 WT protein and mutants. All scale bars: 5 μm.

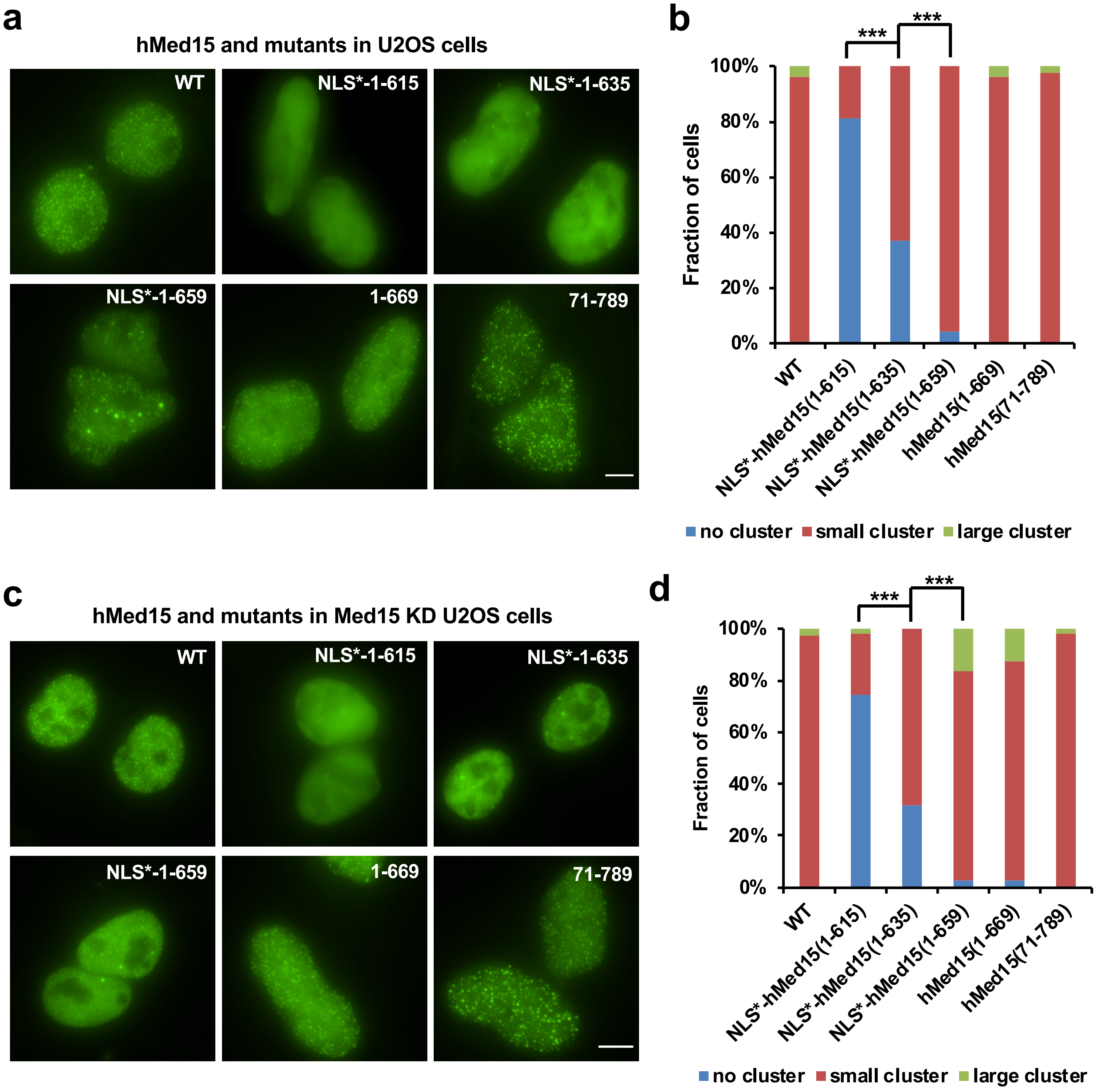

Fig. S13 Formation of nuclear condensates by human Med15 and its truncation mutants in U2OS cells. a, c Representative images of wild-type U2OS cells (a) and Med15 knockdown U2OS cells (c) expressing each human Med15 truncation mutant fused to AcGFP at N-terminus. All scale bars: 5 μm. **b, d** Percentages of wild-type U2OS cells (b) or Med15 knockdown U2OS cells (d) that display no nuclear clusters, small nuclear clusters (diameter < 1 μm) and large nuclear clusters (diameter > 1 μm) of human Med15 (WT) protein or human Med15 truncation mutants. The numbers of analyzed cells were 53, 54, 81, 47, 51 and 42 in b, respectively, and were 40, 58, 47, 37, 39 and 49 in d, respectively. Fisher's exact test: $P < 0.001$ (indicated by ***).

Fig. S14

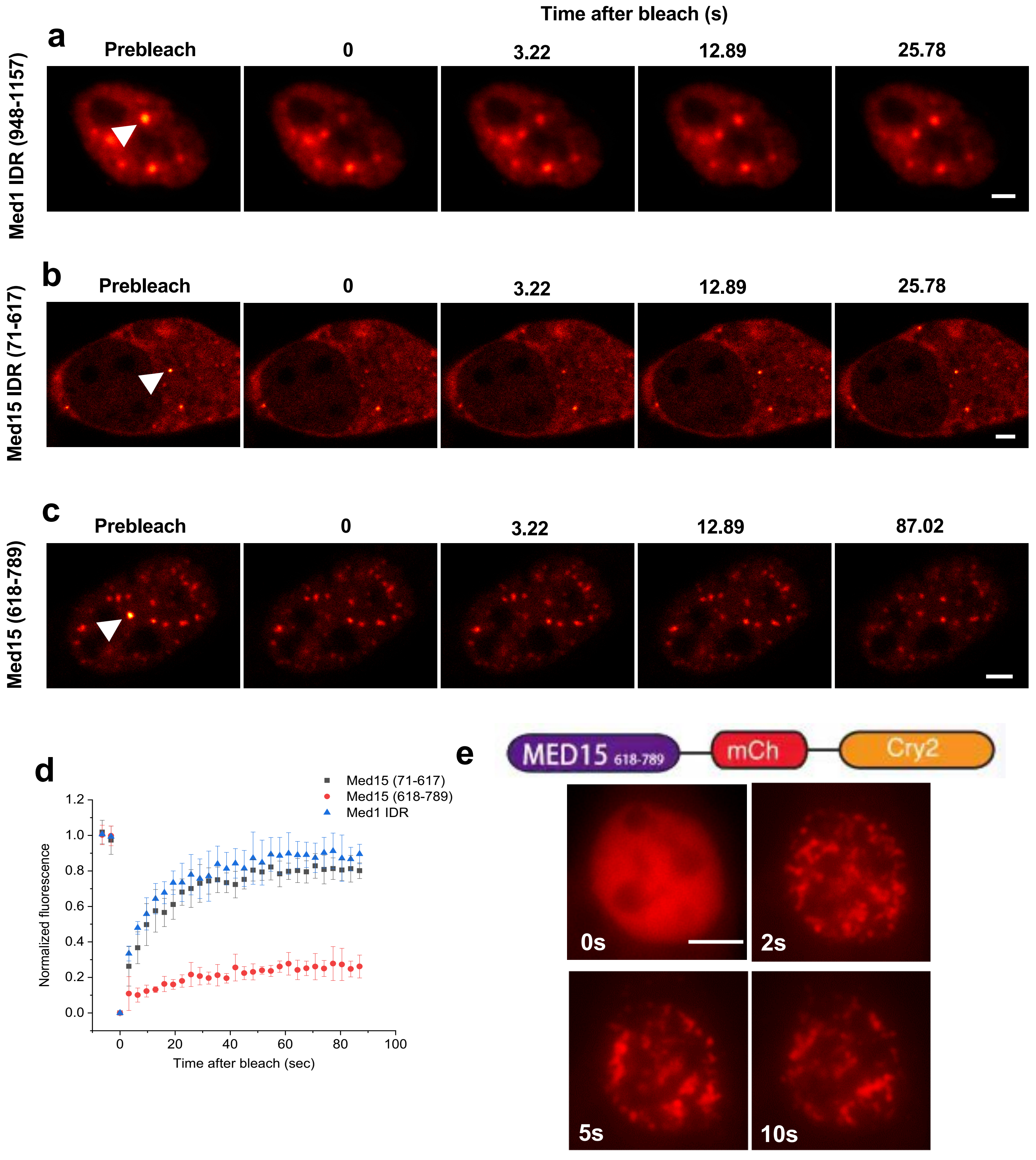

Time after bleach (s)
a
Prebleach
0
3.22
12.89
25.78
Med1 IDR (948-1157)
b
Prebleach
0
3.22
12.89
25.78
Med15 IDR (71-617)
c
Prebleach
0
3.22
12.89
87.02
Med15 (618-789)
d
Med15 (71-617)
Med15 (618-789)
Med1 IDR
Normalized fluorescence
Time after bleach (sec)
e
MED15 618-789
mCh
Cry2
0s
2s
5s
10s

Fig. S14 Dynamics of optodroplets formed by Med1 and Med15 regions. a-c Time-lapse images of NIH3T3 cells expressing the following constructs: Med1-IDR (948-1157)-mCherry-Cry2 (a), NLS*-Med15-IDR (71-617)-mCherry-Cry2 (b) and Med15 (618-789)-mCherry-Cry2 (c) during a FRAP experiment. White arrows indicate the photobleached optodroplets. t=0 s indicates the starting point of photobleaching. The time interval between individual frames was 3.22 s. Scale bars: 2 μm. Similar results were obtained from two independent experiments. **d** Plot of mCherry fluorescence intensity at the photobleached area. FRAP curves were re-normalized to adjust post-bleach fluorescence intensity to 0 for all experiments. Error bars are SEM. Number of cells analyzed were 5, 3, 2 for Med15 (71-617), Med15 (618-789) and Med1 IDR, respectively. **e** Time-lapse images showing the formation of non-spherical optodroplets in NIH3T3 cells by Med15 (618-789)-mCherry-Cry2. t=0 s indicates the starting point of blue light illumination. Time intervals between starting blue light activation and acquiring mCherry fluorescence images are noted on each image. Scale bar: 5 μm.

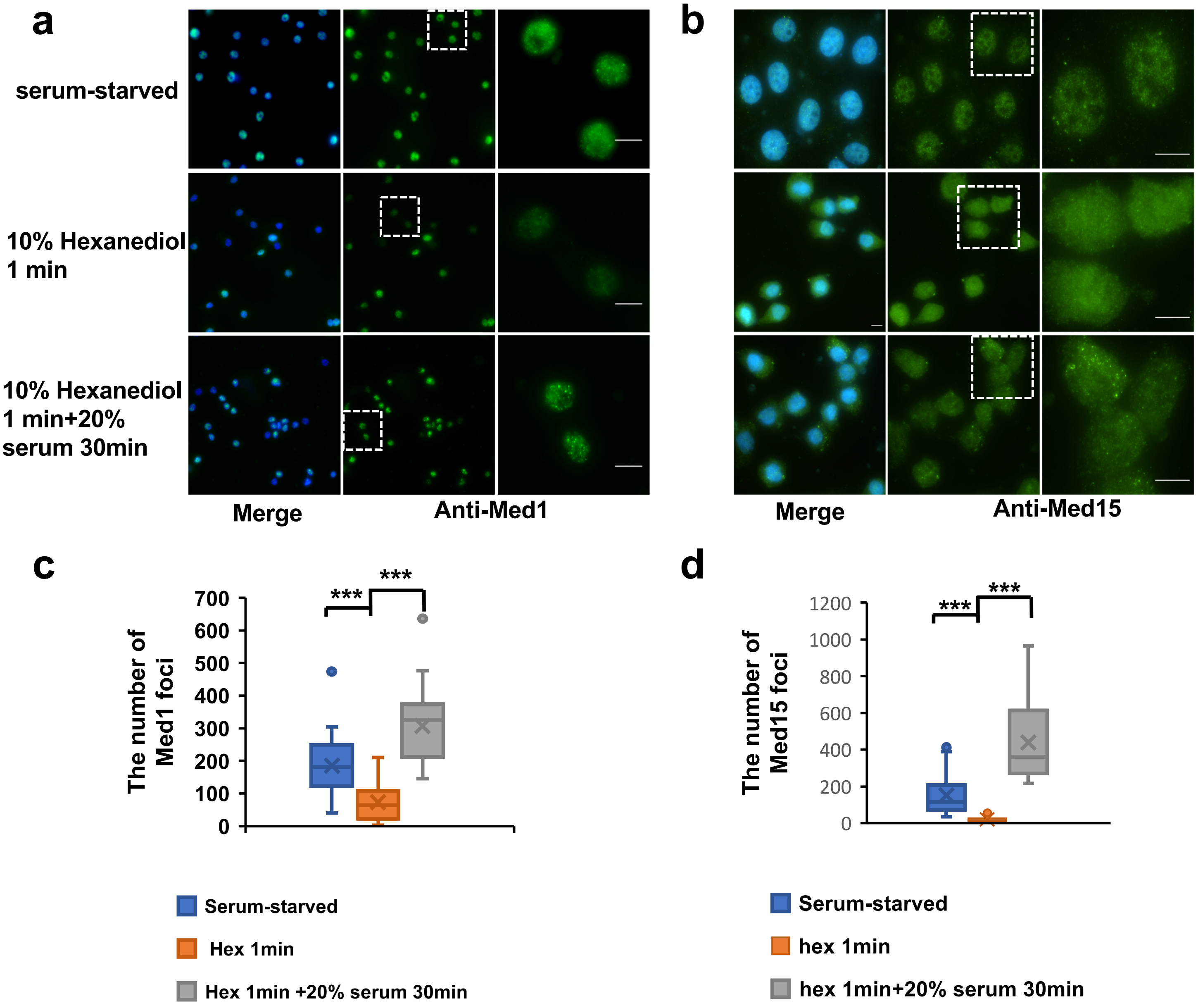

Fig. S15 Response of Med1 and Med15 nuclear foci to 10% Hexanediol treatment and withdrawal in the serum response experiment. a, b Fluorescence images of U2OS cells stained with an anti-Med1 antibody (a) or an anti-Med15 antibody (b) and counterstained with Hoechst33342 (blue). Upper row: serum-starved for 24 h, Middle row: serum-starved for 24 h and treated with 10% Hexanediol for 1 min, Lower row: serum-starved for 24 h, treated with 10% Hexanediol for 1 min and recovered in growth media with 20% serum for 30 min. The right column contains the enlarged anti-Med1 or anti-Med15 immunofluorescence images of the areas marked with white border in the middle column. Scale bars are 10 μm. Similar results were obtained from two independent experiments. **c** The number of Med1 foci in each experimental group described in a (Intensity threshold: 700). The number of analyzed cells were 44, 43 and 52, respectively. **d** The number of Med15 foci in each experimental group described in b (Intensity threshold: 400). The number of analyzed cells were 40, 46 and 31, respectively. Student's t-test: $p < 0.001$ (indicated by ***).

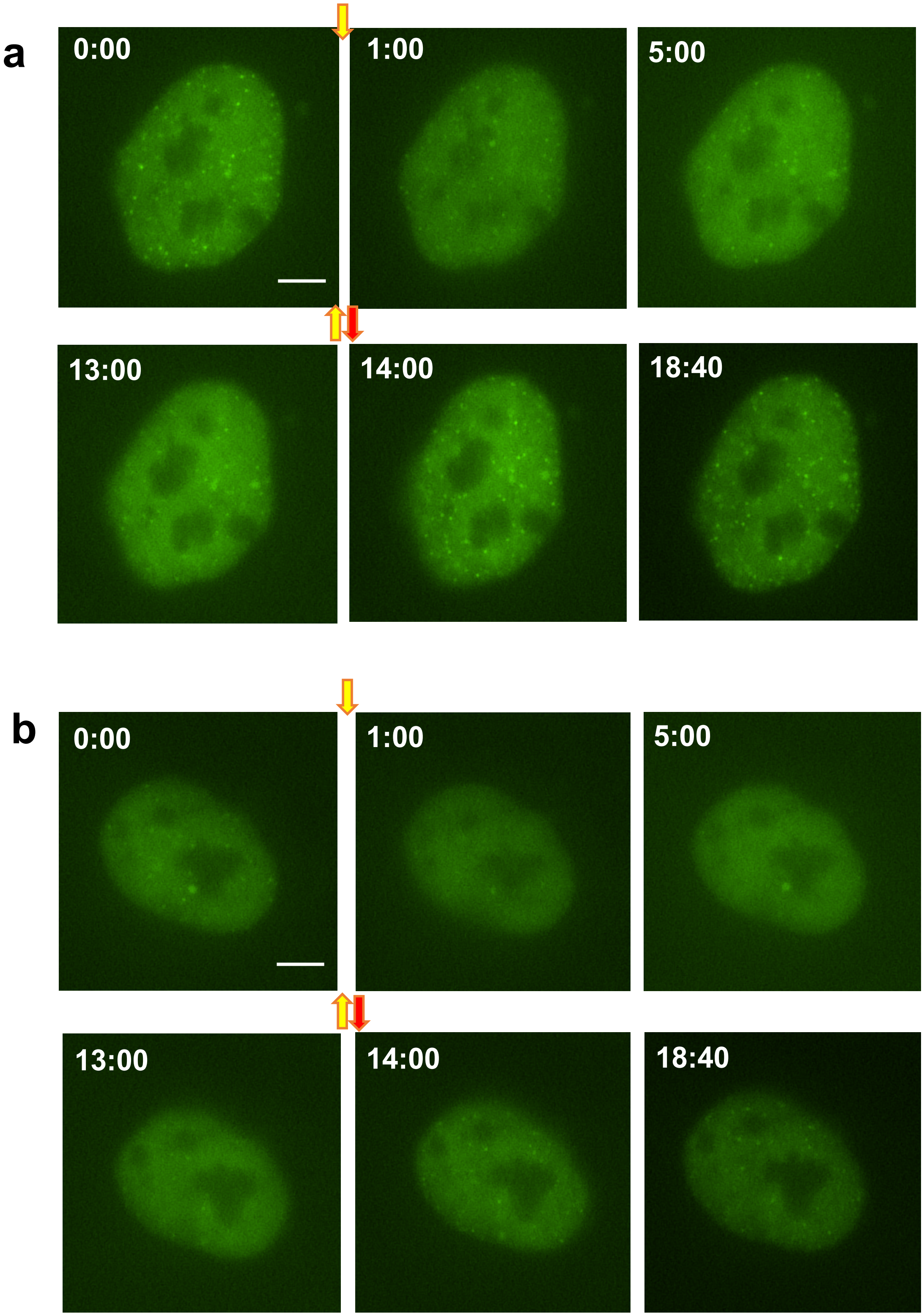

Fig. S16 Time lapse images of serum-starved T24 cells stably expressing GFP-hMed15 upon 0.5% Hexanediol treatment followed by 20% serum stimulation without Hexanediol. 0.5% Hexanediol was added at 1 min and was replaced with fresh growth media containing 20% FBS at 14 min. Noted time points on the images are in mm:ss format. Panels a and b indicate two different cells. Scale bars: 5 μm.

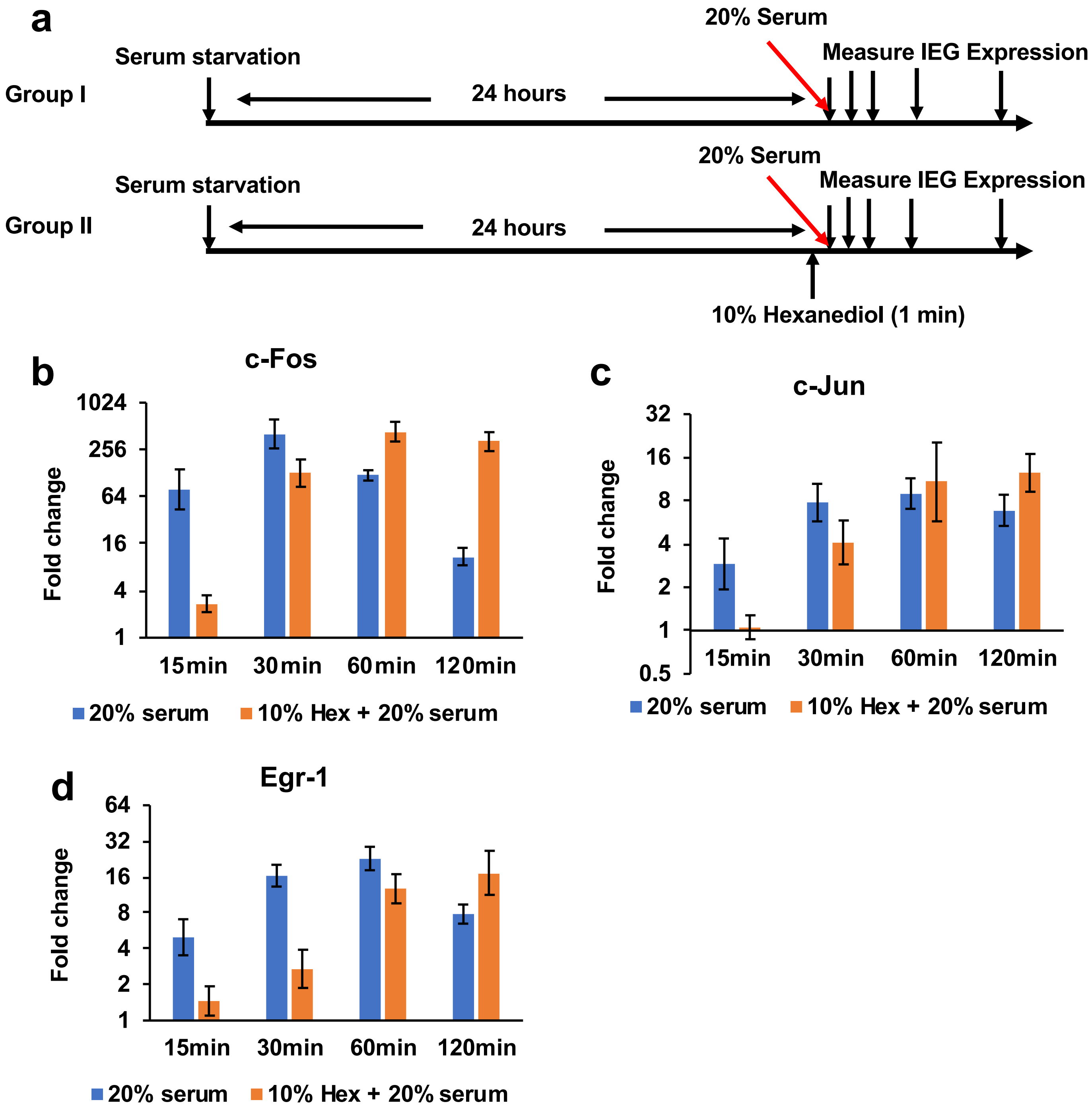

Fig. S17 Effects of 10% Hexanediol treatment on IEG activation during serum response. a The experimental diagram. **b-d** Fold change of c-Fos (b), c-Jun (c) and Egr-1 (d) mRNA expression at 15 min, 30 min, 60 min and 120 min after treatment with 20% serum relative to serum-starved cells in Group I (blue bars) and relative to serum-starved, 10% Hexanediol-treated cells in Group II (orange bars). Four experimental replicates were performed in 15 min (Group I) and 30 min (Group I & II), and three replicates were performed at other time points.

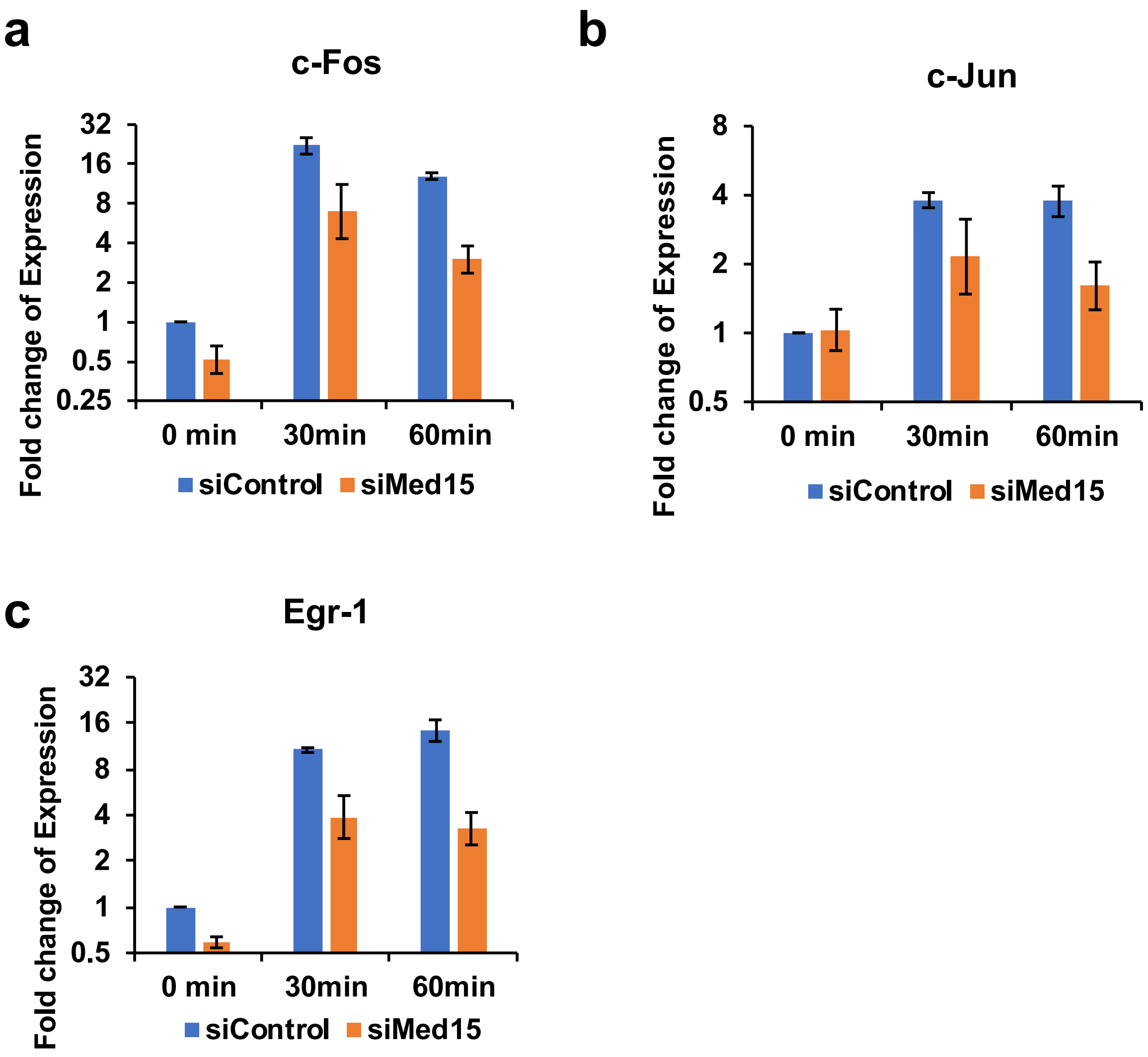

Fig. S18 Effects of Med15 knockdown on IEG activation during the serum response in U2OS cells. a-c Fold change of *c-Fos* (a), *c-Jun* (b) and *Egr-1* (c) expression in U2OS cells transfected with control siRNA (blue bars) or Med15 siRNA (orange bars) in serum-starved cells before treatment (0 min) and after 30 min or 60 min treatment with 20% FBS. In each plot, data were normalized to gene expression levels before serum stimulation in siControl cells. *GAPDH* mRNA expression was used for internal controls. Three experimental replicates were performed and data were presented as mean ± SEM.

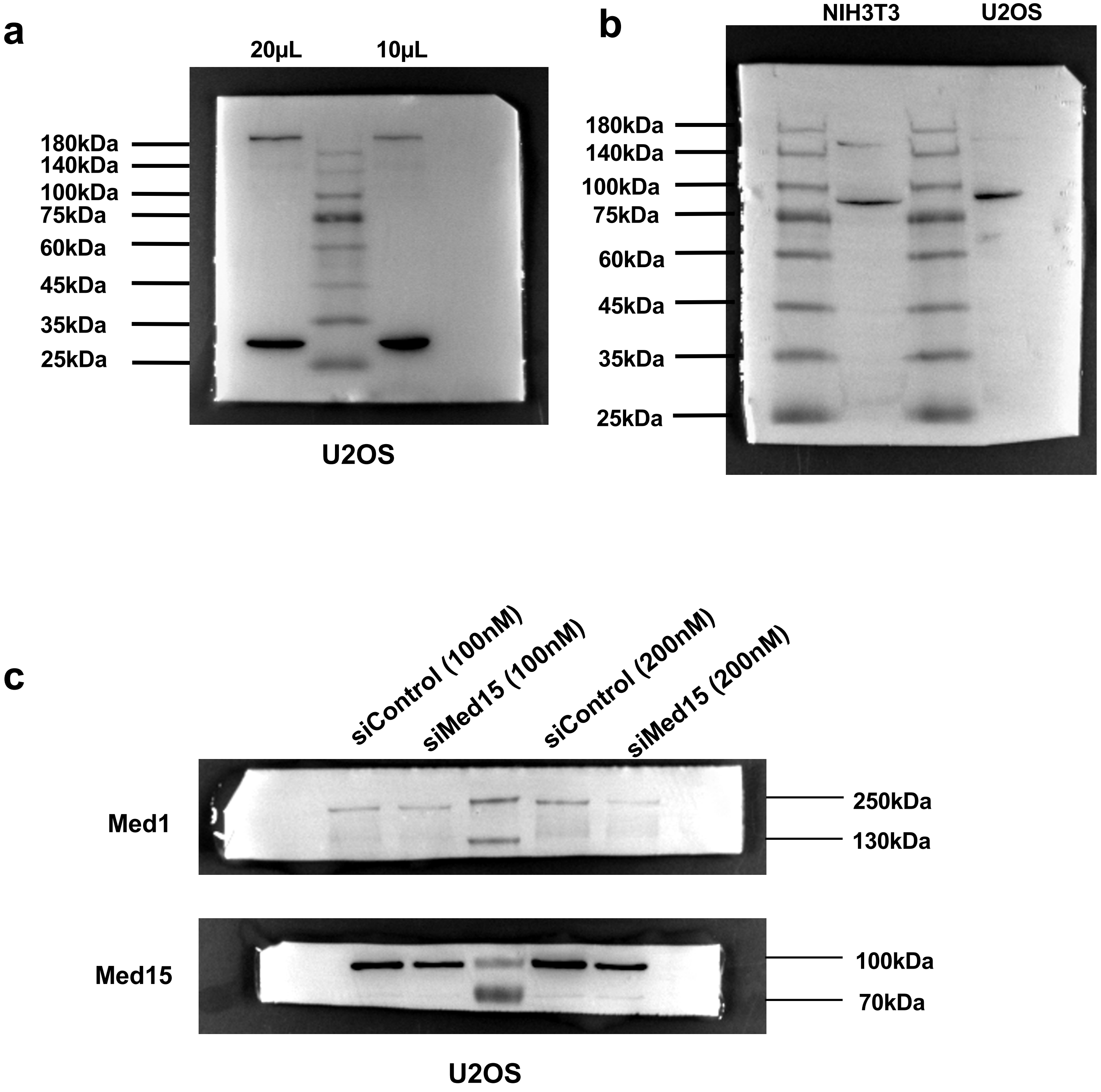

Fig. S19 Original western blot images. a Image of anti-Med1 blot from U2OS cells. **b** Image of anti-Med15 blot from U2OS and NIH3T3 cells. **c** Image of anti-Med1 and anti-Med15 blot from U2OS cells treated with control siRNA and Med15 siRNA at specified concentrations.
