# Supplementary material for: Formation of nuclear condensates by the Mediator complex subunit Med15 in mammalian cells
Source: BMC Biol. 2021 Nov 17;19:245. doi: 10.1186/s12915-021-01178-y (PMC8597291; doi:10.1186/s12915-021-01178-y)
Supplement: Supplementary file 6 — Additional file 6: Table S1-S4.Table S1 Primer sequences used to clone cDNA and mouse Med15 truncation mutants. Table S2 Primer sequences used to clone human Med15 and truncation mutants. Table S3 Primer sequences used to clone the lentiviral vectors. Table S4 Primer sequences used for real-time PCR. [file 12915_2021_1178_MOESM6_ESM.docx]

**Additional file 2. Supplementary tables**

**Table S1** Primer sequences used to clone cDNA and mouse Med15 truncation mutants.

**Table S2** Primer sequences used to clone human Med15 and truncation mutants.

**Table S3** Primer sequences used to clone the lentiviral vectors.

**Table S4** Primer sequences used for real-time PCR.

**Table S1 Primer sequences used to clone cDNA and mouse Med15 truncation mutants.**

|  | primer F (5’-3’) | primer R (5’-3’) |
| --- | --- | --- |
| NPM1 | ggttaagcttcgatggaagattcgatggacatgg | ggttggatccttaaagagacttcctccactgcc |
| DYRK3 | ggttgaattctatgccgaagaagaagcgaaaggtcggaggcacagctcgtgg | ggttggatccctagctaatcagttttggcaatacac |
| Med15-WT | ggttgaattcgatggacgtttcggggcagga | ggttggtaccctaggcagctgagaggcagg |
| Med1-IDR | ggttgaattcggagcaccacagtgggagt | ggttgggccccatgagatcatcatcttcct |
| Med15(100-600) | ggttgaattcgggcatgcctcctcggggc | ggttggtacccatgtcattcttgagcttct |
| Med15(1-617) | ggttgaattcgatggacgtttcggggca | ggttggtaccgtcctgctgtttggttgg |
| Med15(1-636) | ggttgaattcgatggacgtttcggggca | ggttggtaccgttgaagacaggtgaacgg |
| Med15(1-670) | ggttgaattcgatggacgtttcggggca | ggttggtaccatcctcctcaaaccggc |
| Med15(1-680) | ggttgaattcgatggacgtttcggggca | ggttggtaccttgcagcacattgggtatg |
| Med15(71-789) | ggttgaattcgaacaagaaatcccaagcttct | ggttggtaccctaggcagctgagaggca |
| Med15(618-789) | ggttgaattcgctgtgccaaccactccta | ggttggtaccctaggcagctgagaggca |
| Cry2 | ggttctcgagctatgaagatggacaaaaagactatagtttgg | ggttgggcccttatgctgctccgatcatgatc |
| mCherry-N1 | ggttaccggtcgccaccatggtgagc | ggttgcggccgccttgtacagctcgtccatg |
| Med1(948-1157)- mCherry-Cry2 | ggttggtaccatggagcaccacagtgggagt | ggttgggccctggagcctggcttccccc |
| NLS*-Med15(71-617)-mCherry-Cry2 | ctaccggactcagatctcgagatgccgaagaagaagcgaaaggtcaacaagaaatccca | ggatcccgggcccgctgggtaccgtcctgctgtttggttggaaga |
| Med15(618-789)-mCherry-Cry2 | ctaccggactcagatctcgagatgctgtgccaaccactcctagatg | ggatcccgggcccgctgggtaccggcagctgaga  ggcaggcctgg |

**Table S2 Primer sequences used to clone human Med15 and truncation mutants.**

|  | primer F (5’-3’) | primer R (5’-3’) |
| --- | --- | --- |
| Med15-WT | tcgagctcaagcttctgaattcatggacgtttccgggcaa | ggatcccgggcccgcggtaccctaggcggctgagaggcag |
| NLS*-Med15  (1-615) | cgagctcaagcttctgaattcccgaagaagaagcgaaaggtcatggacgtttccgggca | ggatcccgggcccgcggtaccctgctgtttggtcggtggc |
| NLS*-Med15  (1-635) | cgagctcaagcttctgaattcccgaagaagaagcgaaaggtcatggacgtttccgggca | ggatcccgggcccgcggtaccgttgaagacaggtgagcg |
| Med15(1-669) | tcgagctcaagcttctgaattcatggacgtttccgggcaa | ggatcccgggcccgcggtaccatcatcctcaagcctgcg |
| Med15(71-789) | tcgagctcaagcttctgaattcaacaagaaatctcaagct | ggatcccgggcccgcggtaccctaggcggctgagaggcag |

|  | primer F (5’-3’) | primer R (5’-3’) |
| --- | --- | --- |
| pSin-EF2-GFP | cccggacgaattcttcgaaatggtgagcaagggcgccgagc | tgcggatcactagtgctagcctacttgtacagctcatccatg |
| pSin-EF2-GFP-hMed15 | cccggacgaattcttcgaaatggtgagcaagggcgccgagc | tgcggatcactagtgctagcctaggcggctgagaggcaggcc |
| pSin-EF2-TagRFP | cccggacgaattcttcgaaatggtgtctaagggcgaagagctga | tgcggatcactagtgctagctcaattaagtttgtgccccagtttgc |
| pSin-EF2-TagRFP-DYRK3 | cccggacgaattcttcgaaatggtgtctaagggcgaagagctga | tgcggatcactagtgctagcctagctaatcagttttggcaataca |

**Table S3 Primer sequences used to clone the lentiviral vectors.**

**Table S4 Primer sequences used for real-time PCR.**

| Gene | primer F (5’-3’) | primer R (5’-3’) | PCR Product Size |
| --- | --- | --- | --- |
| c-Jun(mouse) | gagaacagactgtcagggct | ccttggcttcagtactcgga | 116bp |
| c-Fos(mouse) | cctactaccattccccagcc | cgtggggataaagttggcac | 107bp |
| Egr-1(mouse) | aaccctatgagcacctgacc | gagaagcggccagtataggt | 130bp |
| GAPDH(mouse) | agaacatcatccctgcatcc | cacattgggggtaggaacac | 110bp |
| c-Jun(human) | gagctggagcgcctgataat | ccctcctgctcatctgtcac | 104bp |
| c-Fos(human) | agactacgaggcgtcatcct | ctggtcgagatggcagtgac | 186bp |
| Egr-1(human) | gtcccatttactcagcggca | atcatgggaacctggaagcc | 157bp |
| Med15(human) | gagtccagatgatcacggaagcc | ggatggagcttgacgggatgg | 102bp |
| GAPDH(human) | ggagcgagatccctccaaaat | ggctgttgtcatacttctcatgg | 197bp |
